# Integrating Transgenic Vector Manipulation with Clinical Interventions to Manage Vector-Borne Diseases

**DOI:** 10.1371/journal.pcbi.1004695

**Published:** 2016-03-10

**Authors:** Kenichi W. Okamoto, Fred Gould, Alun L. Lloyd

**Affiliations:** 1 Department of Entomology, North Carolina State University, Raleigh, North Carolina, United States of America; 2 Yale Institute for Biospheric Studies, Yale University, New Haven, Connecticut, United States of America; 3 Fogarty International Center, National Institutes of Health, Bethesda, Maryland, United States of America; 4 Department of Mathematics and Biomathematics, Graduate Program, North Carolina State University, Raleigh, North Carolina, United States of America; CNRS, FRANCE

## Abstract

Many vector-borne diseases lack effective vaccines and medications, and the limitations of traditional vector control have inspired novel approaches based on using genetic engineering to manipulate vector populations and thereby reduce transmission. Yet both the short- and long-term epidemiological effects of these transgenic strategies are highly uncertain. If neither vaccines, medications, nor transgenic strategies can by themselves suffice for managing vector-borne diseases, integrating these approaches becomes key. Here we develop a framework to evaluate how clinical interventions (i.e., vaccination and medication) can be integrated with transgenic vector manipulation strategies to prevent disease invasion and reduce disease incidence. We show that the ability of clinical interventions to accelerate disease suppression can depend on the nature of the transgenic manipulation deployed (e.g., whether vector population reduction or replacement is attempted). We find that making a specific, individual strategy highly effective may not be necessary for attaining public-health objectives, provided suitable combinations can be adopted. However, we show how combining only partially effective antimicrobial drugs or vaccination with transgenic vector manipulations that merely temporarily lower vector competence can amplify disease resurgence following transient suppression. Thus, transgenic vector manipulation that cannot be sustained can have adverse consequences—consequences which ineffective clinical interventions can at best only mitigate, and at worst temporarily exacerbate. This result, which arises from differences between the time scale on which the interventions affect disease dynamics and the time scale of host population dynamics, highlights the importance of accounting for the potential delay in the effects of deploying public health strategies on long-term disease incidence. We find that for systems at the disease-endemic equilibrium, even modest perturbations induced by weak interventions can exhibit strong, albeit transient, epidemiological effects. This, together with our finding that under some conditions combining strategies could have transient adverse epidemiological effects suggests that a relatively long time horizon may be necessary to discern the efficacy of alternative intervention strategies.

## Author Summary

Despite decades of attempted vector control, several vector-borne diseases remain endemic. Recent high-profile studies suggest that candidate vaccines, particularly for dengue, may be less than completely effective as public health interventions. Nevertheless, the epidemiological consequences of using other novel approaches (e.g., transgenic strategies to reduce or replace vector populations) remain highly uncertain. Faced with unclear prospects of any one strategy succeeding in isolation, there is increasing interest in designing a comprehensive public health response to manage vector-borne diseases. Here we use a relatively simple model to study how combining vaccines, transgenic vector manipulation and antimicrobial medications can facilitate disease management. We explain why the epidemiological consequences for combining strategies are not expected to merely sum their effects. Contrary to the prevailing assumption that comprehensive disease management always yields public health benefits, we find integrating transgenic vector manipulation with clinical interventions can, in some cases, temporarily exacerbate the adverse consequences of any one strategy failing. These results highlight the need for system-specific modeling efforts aimed at assessing whether our conclusions apply to specific vector-borne diseases. We outline the implications for proceeding with public health responses integrating currently available products, as well as assessing their efficacy.

## Introduction

Vector-borne diseases account for a significant share of the global infectious disease burden ([1]), comprising as much as 17% of this burden according to some studies ([2]). They are typically managed by two broad approaches: (i) clinical interventions (such as vaccines or drug treatments), and (ii) vector-based strategies that aim to decrease transmission by reducing the vector density or interfering with the vector’s ability to transmit the disease-causing pathogen. Several authors have described the epidemiological implications of clinical interventions for vector-borne diseases (e.g., [3, 4]), as well as the consequences of transgenic and traditional vector-based control strategies such as insecticide application (e.g., [4–9]).

For several vector-borne diseases (including dengue, malaria and chikungunya), both genetic vector manipulation strategies (which we define broadly as the introduction of novel genetic material into the vector’s germ line—thus including attempts to spread refractory, vertically transmitted endosymbionts such as *Wolbachia* through vector populations—e.g., [10, 11]) and clinical interventions are active research areas ([12–16]). However, no study has explored the potential interplay between transgenic vector manipulation strategies and clinical approaches on epidemiological dynamics. The question is particularly timely, because for several vector-borne diseases (including dengue, malaria and chikungunya), combined approaches are increasingly viewed as necessary for effective disease management (e.g., [3, 16–20]). Combining strategies is not without precedent—in at least one control trial in the Gambia, [21] found that integrating chemoprophylactic anti-malarials with insecticide-impregnated bednets produced a synergistic effect in reducing malaria infection (although not malaria death). Thus, should current genetic vector manipulation methodologies and clinical interventions prove insufficient in isolation, assessing how a multi-faceted strategy combining both approaches could facilitate disease management becomes an especially salient question.

Several authors have modeled the epidemiological implications of clinical interventions for vector- and non-vector-borne diseases (e.g., [3, 22–29]), as well as the effects of traditional and transgenic approaches to reducing vector population sizes on disease incidence (e.g., [4, 5, 7, 30–32]). Relatively few studies have sought to describe the epidemiological effects of introgressing anti-pathogen transgenes into vector populations ([6, 8, 9]). More recently, both empirical and theoretical studies have sought to evaluate the combined effects of vector population reduction using traditional control (e.g., insecticides) and clinical interventions for vector borne disease dynamics (e.g., [33–35]).

By contrast, we know relatively little about the epidemiological impacts of combining genetic vector control with vaccines or antimicrobial medications. For instance, using classical Ross-Macdonald equations for malaria, [6] and [9] investigated the effects of transgenic population replacement strategies on infection prevalence in hosts. They showed that unless complete vector incompetence is achieved population-wide, the ability of a transgenic strategy to suppress a vector-borne disease in areas of intermediate to high transmission is negligible. However, this conclusion depends on the absence of any additional public health interventions to reduce transmission, and is based on an analysis of a particularly highly transmissible pathogen. To our knowledge, no study to date has explored the potential interplay between transgenic vector manipulation strategies and clinical approaches on epidemiological dynamics.

Here, we analyze how different transgenic vector population manipulation strategies can interact with distinct clinical interventions to drive epidemiological dynamics. We compare how effective alternative combinations of transgenic and clinical interventions are at preventing pathogen invasion, as well as how these strategies can reduce incidence and prevalence in disease-endemic situations.

## Models

We base our analysis on a model characterizing the dynamics of a vector-borne disease caused by an infectious pathogen in single vector—single host systems. Vector density may differ across space ([36]), and hosts and vectors may exhibit considerable individual variation in their susceptibility and ability to transmit pathogens (e.g., [37, 38]). These, as well as other sources of heterogeneity, can be important in driving the dynamics of vector-borne infectious diseases (e.g., [39–42]). Nevertheless, we adopt a mean-field approach in order to distill the essential features governing the epidemiological consequences that can result from integrating transgenic vector manipulation and clinical interventions. We see this as an important prerequisite for developing a set of baseline expectations, especially given potential nonlinearities governing the interplay between the different strategies. Such an approach can provide a point of departure for subsequent studies aiming to understand how system-specific sources of heterogeneity (such as spatial structurce—e.g., [43–45], differences among hosts in their attractiveness to vectors—e.g., [46], variability in social constraints that affect the feasibility of deploying an intervention—e.g., [47, 48], and background genetic variation among individual vectors—e.g., [49]) can alter the epidemiological effects of combining qualitatively distinct public health strategies.

The basic dynamics of similar models without interventions have been studied by [50], among others (e.g., [51, 52]). The dynamics of the host-vector-pathogen system are given by:

$$\begin{array}{ccc} \frac{dS}{dt} & = & \left(1-\epsilon \right)b-\psi \frac{VS}{S+I+R}-dS\\ \frac{dI}{dt} & = & \psi \frac{VS}{S+I+R}-\left(d+a+g+\delta \right)I\\ \frac{dR}{dt} & = & \epsilon b+gI+\delta I-dR\\ \frac{dU}{dt} & = & m\left(t\right)F\left(U,V\right)-G\left(t\right)\phi \frac{UI}{S+I+R}-\mu U\\ \frac{dV}{dt} & = & G\left(t\right)\phi \frac{UI}{S+I+R}-\mu V \end{array}$$ (1)

In system (1), the state variables *S*, *I*, *R*, *U* and *V* denote the densities (abundances per unit area) of susceptible hosts, infectious hosts, recovered hosts, susceptible vectors, and vectors infected with the pathogen, respectively. The functions *G*(*t*) and *m*(*t*) describe the effect of transgenic interventions on vector competence and recruitment, respectively, and the parameters *ϵ* and *δ* describe the effects of clinical interventions on epidemiological dynamics. They are described and justified further in later sections. In the absence of any interventions, *δ* = *ϵ* = 0 and *G*(*t*) = *m*(*t*) = 1.

We model transmission between vectors and hosts to be frequency-dependent (i.e., vectors encounter host individuals of a given compartment at rates proportional to the fraction of all hosts in the compartment), which is plausible given the biting behavior of arthropods such as mosquitoes where each vector bites a relatively fixed number of hosts during its lifetime (e.g., [53]). The parameter *ψ* is a transmission constant governing the acquisition of the pathogen from vectors among hosts, and subsumes the vector’s per-capita encounter rates with, and biting rate of, host individuals, as well as the probability of pathogen transmission during a biting event. Susceptible vectors encounter and acquire the pathogen from infectious hosts, becoming infectious at a per-capita rate $G\left(t\right)\phi \frac{I}{S+I+R}$. We ignore vertical transmission and the effects of incubation delays both for simplicity and tractability.

The susceptible host density increases by a constant rate *b* (due to births and immigration), and hosts either die or emigrate at a per-capita rate *d*. For simplicity and tractability, we do not include immigration of infectious or recovered hosts in the modeled system. Infectious hosts suffer an additional per-capita death rate *a*, and, in the absence of clinical interventions, recover at a per-capita rate *g*, after which they are assumed to gain immunity for the duration of the time horizon over which the dynamics of model (1) are analyzed. Thus, our model most readily applies to vector-borne diseases such as dengue or the plague, where a single infection event by a single strain or serotype confers lifelong immunity in the host. We note that some vector-borne diseases for which hosts acquire limited immunity following infection (such as malaria) would require alternative modeling assumptions to those presented here.

We use the widely-applied logistic growth function *F*(*U*, *V*) = (*U* + *V*)(*r* − *k*(*U* + *V*)) to describe vector population recruitment into the epidemiologically relevant life stage (i.e., adult females for mosquito-vectored diseases; [54]). Here, *r* describes the intrinsic per-capita growth rate of the vector population and *k* characterizes density-dependent effects on recruitment. Vectors die or emigrate out of the system at a per-capita rate *μ*. The model assumes that the parameters governing vector demography are not affected by infection with the pathogen, and that all the parameters are constants (Table 1).

**Table 1:** Model parameters and their numerical values.

Parameter	Interpretation	Units	Default values	Reference/notes
*b*	Influx of (susceptible) hosts	hosts ⋅ day^−1^	45.7	Results in an equlibrium host density of 10^6^ without the pathogen
*ψ*, *ϕ*	Transmission constants governing acquisition of pathogen from infectious vectors to susceptible hosts (*ψ*), as well as the acquisition of pathogen from infectious hosts to susceptible vectors (*ϕ*) in the absence of genetic vector manipulation	day ^−1^	0.2	Inferred from type reproductive number^a^
*d*	Host background per-capita mortality rate	day^−1^	$\frac{1}{21900}$	[51, 52]
*a*	Pathogen induced per-capita host mortality rate	day^−1^	0.008549	Based on [67], which reports of a 5% host mortality rate from dengue
*g*	Natural infectious host recovery rate	day^−1^	$\frac{1}{6}$	[51]
*r*	Intrinsic per-capita vector growth rate	day^−1^	0.835	Midpoint of range in [99]
*k*	Density-dependent effects on recruitment	(vectors ⋅ day)^−1^	3.675×10^−7^	Based on an assumption of approximately 2 vectors per host
*μ*	Vector per-capita mortality rate	day^−1^	$\frac{1}{10}$	[44] and [96]
*β*	Parameter governing average vector competence immediately following the onset of the release of transgenic mosquitoes	−	−10	Calibrated so that at *t* = 0, *G*(*t*) = 0.99995 (i.e., very close to 1).
*α*	Parameter governing the decline in vector competence	day^−1^	Varied	−
*ρ*	The number of transgenic males carrying a dominant lethal construct released	−	Varied	−
*ϵ*	Fraction of new hosts vaccinated	−	Varied	−
*δ*	Medication-induced recovery rate	day^−1^	Varied	−

Parameters, their interpretations, units, and default values used in the numerical analysis. Unless stated otherwise, the default values represent the midpoint of the ranges reported in the relevant sources.

^a^ For dengue, the number of secondary host infections arising from a single host infection (the type reproductive number) in the literature has typically been reported to range from less than 1 to greater than 10 (e.g., [51] and [40]). We present results in the main text for a type reproductive number of approximately 4.56.

Aside from incorporating the effects of transgenic manipulation on vector competence or recruitment (and, hence, epidemiological dynamics), the main departure of our model (1) from [50] is that we allow vector populations to potentially be subject to density-dependent regulation, whereas vectors in the model of [50] are subject to no such regulation (instead, the model of [50] assumes exponentially growing host population dynamics and density-independent vector recruitment). System (1) also differs in several important respects from earlier studies ([6, 8, 9]) that explored the epidemiological effects of transgenic population replacement. First, as they aimed to model malaria control, previous studies did not explicitly include the dynamics of an immune, recovered class of hosts. Including this class allows us to compare the epidemiological effects of clinical interventions (such as vaccines) which increase the fraction of immune hosts and hence influence the pathogen transmission rate between hosts and vectors. Second, in contrast to previous models, system (1) does not assume total host population size to be constant. Our model might therefore describe a wider range of systems, particularly those where host demographic and epidemiological dynamics occur on comparable time scales.

S1 Text gives the equilibria of system (1) in the absence of interventions. Important equilibria are the disease-free equilibrium (*S*^⋆^, 0, 0, *U*^⋆^, 0) that will allow us to specify the conditions for the pathogen to invade system (1), and the non-zero equilibria (*S*^⋆^, *I*^⋆^, *R*^⋆^, *U*^⋆^, *V*^⋆^).

We use Eq (1) to analyze and compare how different combinations of transgenic vector manipulation and clinical interventions affect disease management objectives. Below, we describe how we incorporate each of these strategies into our framework.

### Modeling genetic manipulation

#### Population replacement

We model change in average vector competence resulting from the introgression of foreign genetic elements (e.g., transgenic constructs) governing the ability of vectors to become infectious after they acquire the pathogen from hosts. For instance, even though some pathogen particles may be mechanically transmitted to vectors from infectious hosts, a transgenic construct may inhibit within-vector pathogen replication (e.g., [55, 56]), thus preventing a fraction of vectors from becoming infectious. For brevity, we refer to the quantity *G*(*t*) as “relative vector competence”; this function specifies how transgenic vector population manipulation affects vector competence. The functional form of *G*(*t*) can vary according to the specific population replacement strategy employed and the tempo and magnitude of the release schedule. However, we show in S2 Text that a logistic function of the form $G\left(t\right)=\frac{1}{1+\exp(\alpha t+\beta )}$ approximately characterizes the trajectory of the decline in vector competence predicted by several different mechanistic models that explicitly characterize the population genetics governing the spread of anti-pathogen constructs. By varying the shape parameters *α* and *β* (see Table 1 for their biological interpretation), we can quite closely resemble the effects of different proposed population replacement regimes under a wide range of parameter values (S2 Text). We further show in S3 Text that the epidemiological dynamics predicted by a model of *Wolbachia* spread are very similar to the epidemiological dynamics predicted by a model using our logistic approximation to describe the decline in vector competence. Using this logistic function allows us to gain considerable analytical tractability. Thus, we can capture the key effects of a wide range of transgenic manipulation strategies on both vector competence and the epidemiological effects of such interventions, at least in well-mixed systems.

We consider two distinct effects transgenic vector manipulation strategies can have on the trajectories of the ability *G*(*t*) of the vector to become infectious:

- vector competence declines monotonically and remains at very low levels, and
- Failed or abruptly ended transgenic manipulation strategies that result in merely transient reductions in vector competence, which we model as the pathogen developing resistance to the construct after some point *τ*_*R*_ in time. We model this by partitioning the infectious host and vector populations into two subpopulations: those infected with the mutant pathogen resistant to the construct, and those infected with the resident pathogen. For simplicity, we assume that infection by either pathogen subtype confers cross-immunity (which can be reasonable if the resistance allele does not affect proteins recognized by the host’s adaptive immune system), coinfection is negligible, and that the resistant strain’s vector competence does not decline in response to the spread of the construct (i.e., susceptible hosts get infected with the resistant invader at a per-capita rate *ϕV*_*i*_(*t*)/(*S*(*t*) + *I*_*r*_(*t*) + *I*_*i*_(*t*) + *R*(*t*)), where *V*_*i*_(*t*), *I*_*i*_(*t*) are the densities of vectors and hosts, respectively, infectious with the resistant pathogen and *I*_*r*_(*t*) is the density of hosts infectious with the resident pathogen).

We analyze how varying the magnitudes of the constants (*α*, *β* and *τ*_*R*_) governing the speed of population replacement affects epidemiological dynamics.

#### Population reduction

Transgenic vector manipulation can also aim to reduce vector population density. For example, in the dengue vector *Aedes aegypti*, the lethal RIDL transgenic construct acts early in the adult life-stage, before individual vectors become infectious, by preventing female wing muscle development (e.g., [57]). We therefore model transgenic vector population reduction as reducing recruitment into the potentially infectious life stage of the vector by a factor *m*(*t*).

The vector population dynamics that result from transgenic population reduction strategies are relatively consistent across proposed constructs. Typically, as males cannot vector the pathogen, transgenic males carrying a lethal construct are released and reproduce with wild-type females. The resulting offspring do not develop into viable vectors. Thus, the extent to which these strategies reduce the vector recruitment rate depends on the ratio of released, transgenic males to wild-type males in the community. We therefore model the effect *m*(*t*) of the transgenic intervention on recruitment as the probability that a wild-type female vector mates with a wild-type male. We consider two potential outcomes in our analyses:

- vector recruitment into the potentially infectious life stage decreases by a factor ($m\left(t\right)=\frac{U+V}{U+V+\rho }$), where *ρ* is the average density of transgenic males encountered by females (e.g., [57, 58]). Because transgenic males are assumed to be reared separately from wild vectors, they do not directly affect the strength of density-dependent recruitment. If the number of transgenic males in the system reaches an equilibrium quickly and the release numbers are constant over time, then *ρ* is the number of transgenic males released per unit time divided by the per-capita mortality rate of those males. We model the case where matings between transgenic males and wild-type females cannot produce viable offspring of either sex. As in [57], we assume the sex-ratio between wild-type male and wild-type female vectors is one-to-one in the vector population (and thus the density of wild-type males can be modeled to be sufficiently close to the density (*U*+*V*) of wild-type females).
- the vector recruitment rate decreases as described above until some point *τ*_*m*_ in time, after which it returns to pre-control levels (i.e., *m*(*t*) = 1). This might occur if, for instance, vector density falls below levels detectable in field surveys and a release program is terminated.

We highlight that when the number of transgenic mosquitoes carrying a lethal construct that are released into the environment over time is constant (e.g., [59]), the ratio of transgenic vectors carrying a lethal construct to the standing wild-type mosquito population grows. Consequently, the per-capita growth rate of vectors decreases as transgenic vector control progresses. This dynamic differs from some traditional control strategies (e.g., insecticide spraying), where the per-capita demographic rates (e.g., mortality) do not depend on the abundance of vectors.

### Modeling the epidemiological consequences of clinical interventions

We model two potential clinical interventions: a program based on vaccination of new hosts, and one based on the administration of antimicrobial medications to infectious hosts. We assume a fixed fraction *ϵ* of new hosts becomes successfully vaccinated, after which these hosts are no longer capable of becoming infectious. Similarly, we model a situation where infectious hosts can acquire and successfully treat their infections with antimicrobials at a rate *δ*. The numerical values of *δ* and *ϵ* encompass both the extent to which the intervention covers individuals in the targeted population, and the per-host efficacy of each intervention. For instance, a vaccine that is only partially effective in protecting susceptible hosts from infection by the pathogen could have a low value of *ϵ*, even if a large number of new susceptible hosts are vaccinated (e.g., [60] and [61]). Similarly, an antimicrobial that may be highly effective on a single infectious host individual may nevertheless have a low *δ* if few infectious hosts take the drug (e.g., due to a high proportion of cases going undiagnosed or the drug being scarce).

## Results

Ideally, combining vaccination, medication treatment, and genetic vector manipulation strategies should facilitate two epidemiological objectives. First, it should be able to prevent the pathogen from invading if it is locally absent. Second, such efforts should reduce the long-term density of infected individuals in the host population, even if the vector-borne disease may already be endemic. Using a combination of analytical and numerical modeling approaches for system (1), we assess and compare the interplay between the different disease management strategies according to how well they satisfy these two criteria.

### How does combining strategies prevent a vector-borne pathogen from invading?

We consider the community to be robust to invasions by the vector-borne disease if the disease-free equilibrium is locally stable. We ask how the different strategies affect the local stability of the disease-free equilibrium. Box 1 describes the basic and effective type reproductive numbers for system (1), which describes the average number of secondary cases in a given type of organism that harbors the pathogen arising from a single infectious individual of the same type (here, hosts and vectors). Box 1 briefly summarizes how the various intervention strategies can affect this value in our model.

Under biologically realistic conditions, i.e., all parameters in Eq (1) are positive and *ϵ* ≤ 1, the pathogen cannot invade whenever

$$\begin{array}{c} \frac{d(1-\epsilon )\phi G(t)(rm(t)-\mu )\psi }{bk(a+d+g+\delta )\mu m(t)}<1. \end{array}$$ (2)

We highlight some key implications of inequality Eq (2). First, whenever transgenic vector population reduction lowers vector recruitment below the per-capita vector recruitment rate scaled by its background mortality rate (i.e., *m*(*t*) < *r*/*μ*), then the left hand side of condition (2) is less than zero, implying successful pathogen suppression. Provided the reduction *m*(*t*) in recruitment achieved through transgenic population reduction does not exceed *r*/*μ* (so that the quotient in condition (2) is positive), increasing the rate *δ* at which antimicrobial medication removes infectious hosts has the greatest effect on preventing pathogen reinvasion when *δ* is small. Once the epidemiological effect of the antimicrobial medication is already large, increasing this value has a diminishing effect on preventing pathogen invasion. Second, condition (2) also implies that the necessary reduction *G*(*t*) in vector competence achieved through transgenic manipulation depends non-linearly on the fraction *ϵ* of hosts vaccinated. Rearranging the terms in Eq (2), it is apparent that when $\phi G\left(t\right)<\frac{k\mu bm(t)(a+d+\delta +g)}{d(1-\epsilon )\psi (rm(t)-\mu )}$ the pathogen cannot become established. Hence, when the vaccination fraction is low (close to zero), condition (2) provides a threshold reduction in vector competence (or level of population replacement) necessary for successful disease suppression. As the vaccination fraction increases towards full coverage, there is a rapid reduction in the decline *G*(*t*) in vector competence necessary to prevent the pathogen from increasing when rare.

**Box 1.**

The average number of secondary host infections arising from a single host infection at day *t* is given by the effective type reproductive number

$$\begin{array}{c} T_{E}=\frac{\psi S(t)U(t)\phi G(t)}{\mu \left(a+d+\delta +g\right){\left(I(t)+R(t)+S(t)\right)}^{2}} \end{array}$$ (3)

(e.g., [62, 63]).

In a naïve host and vector population (where *I*(*t*) = 0), the type reproductive number *T*_*R*_ for Eq (1) can be obtained by determining the disease-free equilibrium densities of the different compartments (S1 Text) and then calculating the expected number of cases among hosts or vectors arising from a single infected individual of the same type. For model (1), Eq (3) implies that the type reproductive number at the disease-free equilibrium is given by

$$\begin{array}{c} T_{R}=\frac{d(1-\epsilon )\phi G(t)(rm(t)-\mu )\psi }{bk(a+d+\delta +g)\mu m(t)}. \end{array}$$ (4)

With appropriate reinterpretation of the parameters, in the absence of any clinical or transgenic interventions, at the disease-free equilibrium this expression is equivalent to the expression of *R*_0_ used for the Ross-Macdonald model (e.g., [64]). We note that *T*_*R*_ < 1 whenever condition (2) in the main text is satisfied.

Eq (4) implies that any public health strategy which reduces the ability of vectors to infect hosts (e.g., transgenic population replacement), the recruitment of susceptible vectors (e.g., transgenic population reduction), or increases the recovery rate of infectious hosts (e.g., medication treatment) lowers *T*_*R*_. We note that in our model, if we characterize vaccination as reducing the ability *ψ* of hosts to acquire the pathogen from the vector, it has a linear effect on reducing the type reproductive number just as reducing *G*(*t*) linearly lowers *T*_*R*_.

As Eq (1) is perturbed and the densities of susceptible, infectious and recovered hosts as well as susceptible and infectious vectors vary over time, it becomes difficult to obtain analytical expressions for the rate at which secondary infections appear, necessitating numerical exploration of the dynamics of system (1) during the transient stages. We characterize how the effective type reproductive number changes in our numerical analyses.

For transgenic interventions aiming at population replacement, we assume there is no feedback (e.g., reactive control measures) from the epidemiological dynamics to the transgenic manipulation strategies. Thus, system (1) and condition (2) permit us to explicitly evaluate the time (*t*_*s*_) necessary for the two kinds of transgenic vector manipulation programs to prevent the pathogen from invading. Indeed, our use of the logistic function *G*(*t*) to capture the essential dynamics of transgenic population replacement facilitates deriving an analytic expression for *t*_*s*_. We note also that for the case involving population reduction, we were able to solve for the total vector population size as an explicit function of time. This enables using Eq (3) to calculate *t*_*s*_, provided the host population is at demographic equilibrium. Table 2 summarizes how the effects of the clinical interventions on *t*_*s*_ depend on the trajectories and nature (population reduction vs. replacement) of transgenic vector manipulation.

**Table 2:** Time *t*_*s*_ to suppression.

Intervention strategy	Time *t*_*s*_ until suppression is feasible	Implications for integrated disease management
Population replacement	$\frac{\beta +\log(\frac{1}{1-k\tilde{U}})}{\alpha }$	Increasing the rate of population replacement (*α*) has an accelerating, nonlinear effect on the time to feasible suppression, while both increasing the medication induced recovery rate or vaccination fraction have a saturating effect on reducing the time to suppression.
Population reduction	$\frac{1}{4\mu \sqrt{4\rho k\mu -{\left(r+\mu \right)}^{2}}}{[2\left(r\mu \right)\left[{\text{tan}}^{-1}\left(\frac{\left(r-\mu \right)}{\sqrt{4\rho k\mu -{\left(r+\mu \right)}^{2}}}\right)-{\text{tan}}^{-1}\left(\frac{z}{\sqrt{4\rho k\mu -{\left(r+\mu \right)}^{2}}}\right)\right]+\sqrt{4\rho k\mu -{\left(r+\mu \right)}^{2}}\left(-2\log\left(\tilde{U}\right)+2\log\left(U^{⋆}\right)-\log\left(\rho \mu \right)+\log\left(\rho \mu +U^{⋆}z\right)\right]}^{a}$	When the vaccination fraction and the medication induced recovery rate are small, increasing the number of transgenic males released has a rapidly diminishing effect on reducing the time to disease suppression.

The effects of integrating transgenic vector manipulation under different evolutionary trajectories with clinical interventions on the stability of the disease-free equilibrium (i.e., suppression) of system (1). The results are based on the derivations in S1 Text. For transgenic population reduction, we present results for the subset of outcomes that, for the duration of the program, do not reduce the recruitment rate below the critical threshold *r*/*μ* at which point population elimination can occur (see the main text for details). Our deterministic, continuous-state model precludes the disease-free equilibrium from being stable following unsustainable transgenic vector manipulation. Thus, we only present results for transgenic vector manipulations that reduce long-term vector competence or recruitment.

^a^ Here, $\tilde{U}=\frac{b(a+d+g+\delta )\mu }{d(1-\epsilon )\phi \psi }$ is the density of susceptible vectors below which the type reproductive number *T*_*R*_ < 1 in the presence of ongoing clinical interventions, and $z=\mu +2k\tilde{U}-r$. The expression for the equilibrium density *U*^⋆^ for susceptible vectors before the transgenic population reduction program begins is given in S1 Text.

Our key result is that depending on the type of transgenic vector manipulation, the interplay between clinical interventions and how the transgenic manipulation affects vector populations can reduce *t*_*s*_ in different ways. For instance, if transgenic manipulation aims at population replacement, the time *t*_*s*_ to suppression is a function of the ratio of the logarithm of one minus the vector density ($\tilde{U}$) necessary to cause suppression in the presence of clinical interventions (scaled by the strength of density-dependent recruitment), to the shape parameter *α* which governs the rate at which population replacement occurs (Table 2). By contrast, there can be multiplicative and additive effects between the reduction *ρ* in recruitment caused by transgenic manipulation and the vector density ($\tilde{U}$) below which suppression becomes feasible when clinical interventions are implemented (Table 2). Furthermore, accelerating transgenic population replacement (increasing *α*) consistently shortens the time until successful suppression, whereas increasing the magnitude *ρ* of transgenically-induced population reduction can, in certain regions of parameter space, have no effect on reducing *t*_*s*_ further depending on the magnitude of the vaccination fraction and medication induced-recovery. Note, for instance, that if *ρ* is already large, then further increasing *ρ* will not reduce *t*_*s*_ in Table 2 as much as increasing *ρ* when *ρ* is initially small. These results illustrate that although both transgenic population reduction and transgenic population replacement ultimately aim to reduce pathogen transmission, they may exhibit subtle differences in how they interact with clinical interventions to facilitate disease suppression.

### When can combining strategies reduce incidence when the vector-borne disease is already endemic?

One of the most compelling reasons for combining strategies is to reduce the number of new infections after the pathogen becomes established. We describe the reduction $\hat{q_{P}}$ in incidence over a time horizon *T*_*H*_ of a vector-borne disease management strategy *P* as

$$\begin{array}{ccc} \hat{q_{P}}\left(T_{H}\right) & = & \frac{\int_{0}^{T_{H}}G\left(t\right)\phi \frac{S_{P}\left(t\right)V_{P}\left(t\right)}{S_{P}\left(t\right)+I_{P}\left(t\right)+R_{P}\left(t\right)}dt}{\int_{0}^{T_{H}}\phi \frac{S_{A}\left(t\right)V_{A}\left(t\right)}{S_{A}\left(t\right)+I_{A}\left(t\right)+R_{A}\left(t\right)}dt}, \end{array}$$ (5)

where the numerator describes the total number of cases, or cumulative incidence, over the time period from 0 to *T*_*H*_ in the presence of a disease management strategy, and the denominator describes the same quantity in the absence of any intervention.

While assessing the reduction in incidence requires simulating the dynamics of system (1), the large number of parameters and strategy combinations make an exhaustive exploration of the parameter space difficult. Therefore, we adopt previously published parameter values for the human-*Aedes* mosquito-dengue system to facilitate our numerical analyses (Table 1). The human-*Aedes* mosquito-dengue system provides an attractive case study for comparing the impact of different strategies on epidemiological dynamics for several reasons. First, there is a relatively large literature on using deterministic, continuous-time models to study dengue epidemiology, at least when compared to some other vector-borne disease systems (reviewed in, e.g., [52]). This provides some degree of continuity to previous work. Second, dengue infections result from vector-borne transmission from one human host to another human host ([65, 66]), allowing us to focus on a single host species (although, for simplicity and as a first step, we do not model multiple dengue serotypes in simultaneous circulation in the system or coexisting potential dengue vectors). Finally, several vaccines, anti-viral medications and transgenic vector manipulation strategies have been proposed for dengue (e.g., [12, 55, 67] and [11]). Field trials are ongoing for both transgenic population reduction and replacement (reviewed in [68]). Trials using transgenic population reduction have aimed at assessing the dispersal and survivorship of transgenic mosquitoes in field settings (e.g., [69]), the ability of transgenic males to effectively compete with wild-type males for mates ([70]), and the magnitude of vector population reduction attributed to the release of transgenic mosquitoes ([71]). Field trials for population replacement in *Ae. aegypti* have focused on attempts to spread *Wolbachia*. Thus far, these studies have shown that *Wolbachia* can indeed become established in the field ([11]) and that introgressed *Wolbachia* could retain refractoriness over relatively short time scales ([72]). However, for both transgenic population reduction and replacement, assessments of their epidemiological effects are only beginning. As vaccines have not yet proven completely effective in eradication ([16, 73–75]), dengue presents a natural case study for evaluating the effectiveness of combining clinical interventions with transgenic manipulation.

Except where we state otherwise, we follow [76] in modeling the impact of the intervention strategies over time horizons of *T*_*H*_ ∈ (0,10] years, which corresponds to a reasonable time-span for public health decision makers ([77]). Previous studies using biologically detailed models of urban *Ae. aegypti* populations have shown this time frame to be sufficient to permit anti-pathogen constructs to reach fixation ([78, 79]). However, using different time horizons may alter the interpretation of some of our results concerning transient dynamics. In particular, although some strategies may exhibit similar reductions in cumulative incidence over a given time horizon, they can vary in their ability to prevent pathogen reinvasion. Under longer time horizons, strategies unable to keep pathogens suppressed will exhibit higher cumulative incidence than strategies robust to pathogen reinvasion. Thus, where applicable, we also assess how combining transgenic manipulation with clinical interventions can affect the disease-endemic equilibrium.

We consider how the interplay between transgenic manipulation strategies and clinical interventions can facilitate reductions in incidence under two basic scenarios:

- How can improving transgenic population reduction or replacement compensate for a less effective clinical intervention?, and,
- How can clinical interventions be best combined with transgenic vector manipulation that only temporarily lowers vector competence or recruitment?

For simplicity and tractability, we assume all intervention strategies begin when system (1) is at the endemic equilibrium. Numerical analyses were carried out using the deSolve and rootSolve packages in R ([80, 81]; S5 Text) and Mathematica ([82]).

### Combining sustainable transgenic population manipulation with partially effective clinical interventions

**Fig 1:**
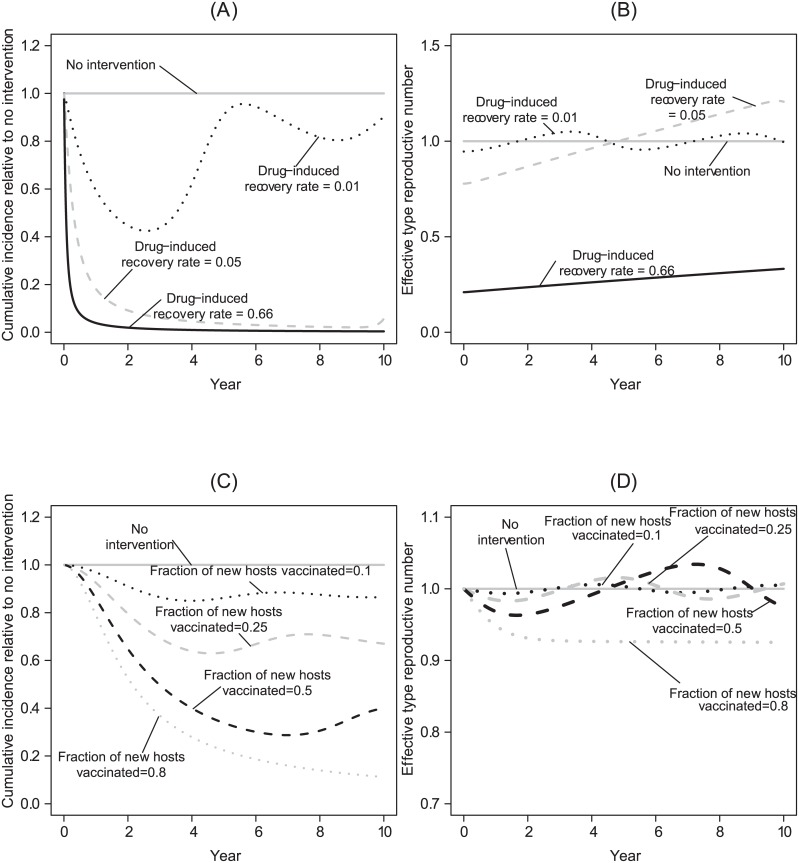
Illustrative time series for the effects of applying a single clinical intervention strategy. Here, and in subsequent figures, in the absence of any intervention the type reproductive number *T*_*R*_ at the disease-free equilibrium is ≈ 4.56; note, however, that at the disease-endemic equilibrium, the effective type reproductive number is one. Here, and in subsequent figures, the solid grey line represents the total number of cases or the effective type reproductive number in the absence of any management strategy. (A) The effects of an antimicrobial medication that renders infectious hosts recovered at different rates on the total number of cases. The natural recovery rate is 1/6 ≈ 0.17 per day. We highlight that even a very weak effect from antimicrobial medications (0.01; approximately 5% of the background natural recovery rate) can cause large transient fluctuations at the disease-endemic equilibrium. The type reproductive number at the disease-free equilibrium is below one when the medication-induced recovery rate is above 0.63 per day. (B) The effects of an antimicrobial medication with different recovery rates on the effective type reproductive number at a given point in time. (C) The effects of vaccinating a fraction *ϵ* of newborns on the total number of cases and (D) the effective type reproductive number at a given point in time. In this, and in subsequent figures, the cumulative incidence relative to no intervention over time is defined as the running aggregate $\hat{q_{P}}\left(t\right)$ (see the main text for details).

All successful strategies reduce the number of secondary host infections (*T*_*R*_, the type reproductive number) below one. However, each strategy used in isolation can prove ineffective for different reasons. For instance, Fig 1 shows how an ineffective clinical intervention that relies on administering medications to infectious hosts can initially reduce the number of secondary infections among hosts below one, but the density of susceptible hosts accumulates because there are fewer infectious hosts. Eventually, sufficient susceptible hosts become available to permit the effective type reproductive number to exceed one (Fig 1B), signaling an end to a “honeymoon period” (e.g., [83, 84]). In contrast to the other approaches, when used in isolation, even relatively effective vaccination strategies do not cause a strong, immediate reduction in the total number of cases (e.g., Fig 1C v. Fig 1A). Thus, while such a strategy can eventually keep the effective type reproductive number below one, the initial effect of a vaccination program may appear modest in comparison to other interventions (Fig 1D). This is because we model vaccination strategies to target incoming susceptible hosts (i.e., newborns and immigrants; model 1). Hence, the time-scales over which their epidemiological effects are realized can differ from the use of antimicrobials or transgenic manipulation. The ability of vaccination that targets new hosts to lower the total number of cases over the time-horizon of interest may therefore be limited in comparison to the other strategies.

**Fig 2:**
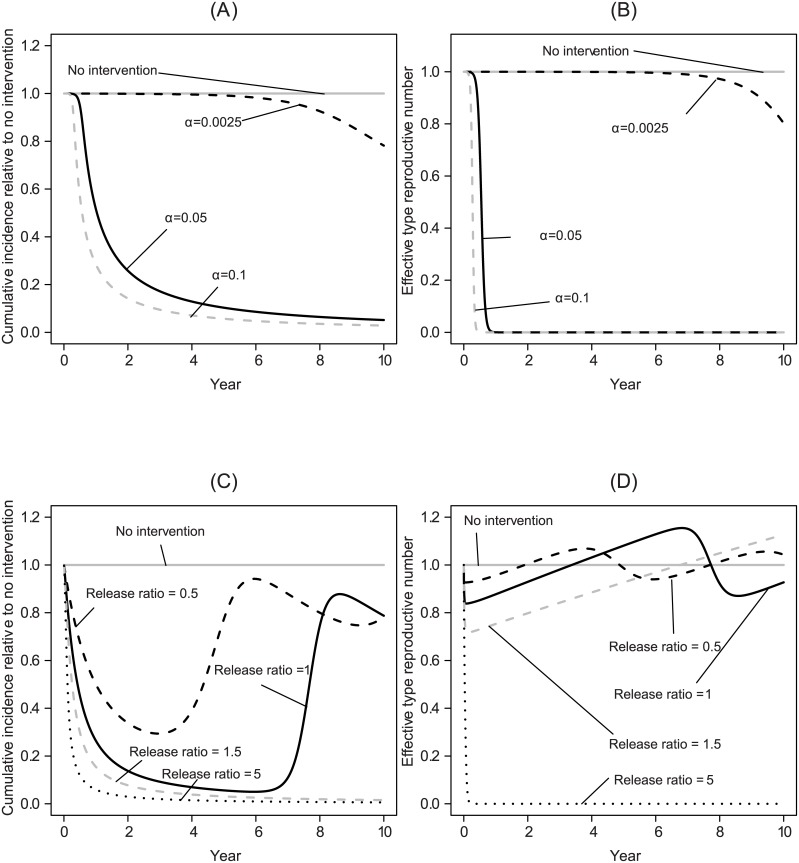
Illustrative time series for the effects of applying only a transgenic vector manipulation strategy. In this, and in subsequent figures, *β* = −10, which corresponds to *G*(0) = 0.99995. (A) The effects on the total number of cases (i.e., cumulative incidence) up until time *t* for transgenic population replacement where the parameter *α* reflects how quickly a vector competent population is replaced through transgenic vector manipulation. A value of *α* governing the decline in vector competence of 10^−1^ per day corresponds to a program that takes approximately 0.4 years to reduce vector competence by 99%, while a value of 2.5 × 10^−3^ corresponds to a program that takes approximately 16 years to reduce vector competence by 99%. (B) The effects on the type reproductive number over time of the different transgenic population replacement strategies. (C) The effects on the total number of cases of transgenic vector manipulation that reduces recruitment of vectors across release ratios. We note that if the transgenic strategy acts on vector recruitment, its effects on vector poulation size are mediated by the effects of other demographic processes (such as density-independent mortality and density dependence). Whereas, when the transgenic strategy aims at population replacement, the effects of the transgenic strategies are proportional to reductions in vector competence. (D) The effects on the type reproductive number over time of the different transgenic population reduction strategies.

When large reductions in vector competence can be maintained, pathogen transmission also remains very low, preventing pathogen reinvasion even if the density of susceptible hosts subsequently increases (Fig 2A and 2B). Similarly, large reductions in vector recruitment can sustainably reduce incidence. However, if transgenic manipulation cannot reduce vector recruitment to very low levels, this will ultimately fail to keep the pathogen suppressed, allowing the type reproductive number to recover and increase above one as susceptible hosts accumulate in the system (Fig 2C and 2D).

**Fig 3:**
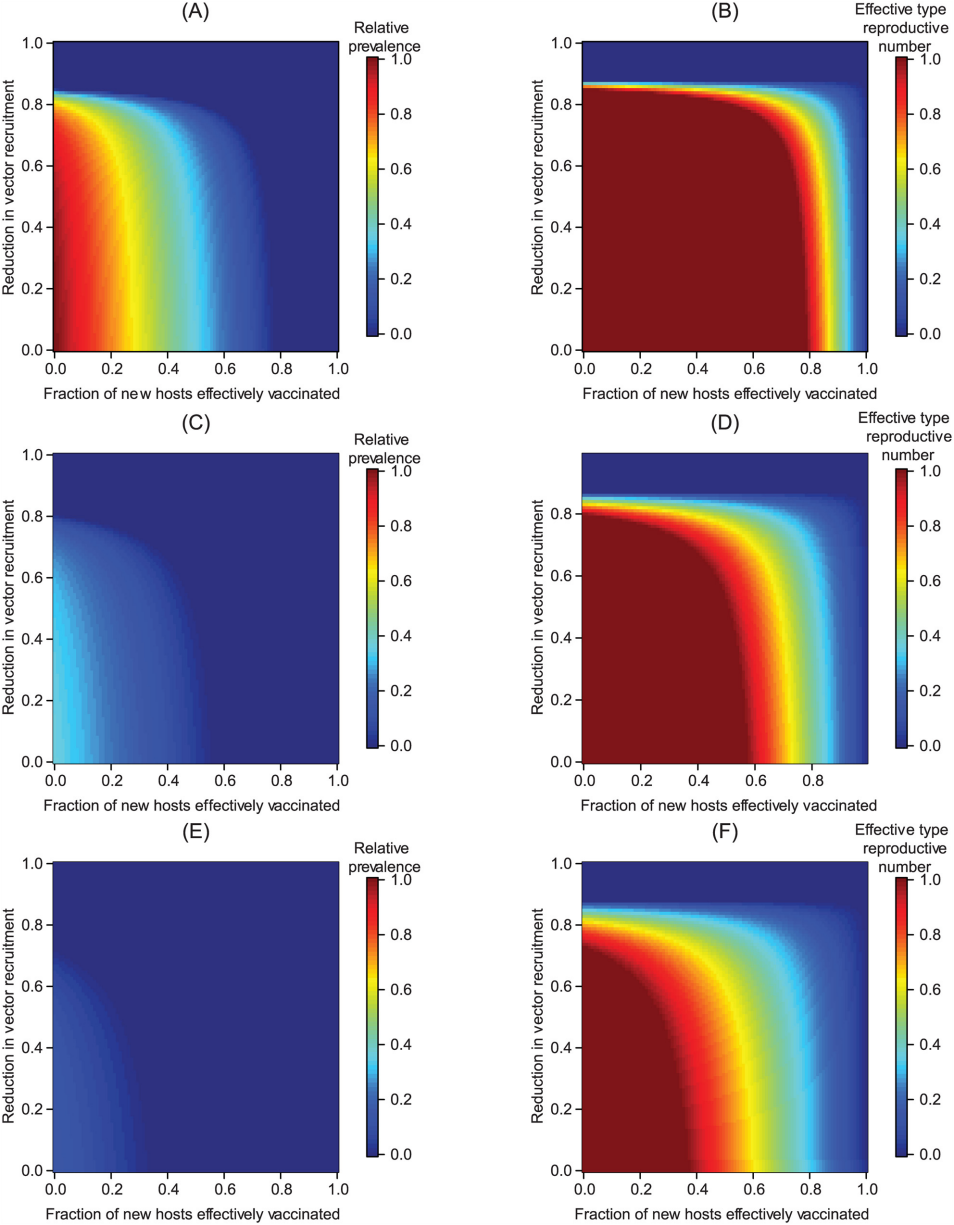
The long-term epidemiological effects of integrating clinical interventions with transgenic population reduction. (A) The prevalence (fraction of hosts infectious) at equilibrium after combined vaccination and transgenic population reduction relative to prevalence before the interventions begin, and (B) the corresponding effective type reproductive number at the equilibrium in (A) in the absence of an antimicrobial medication. (C-D) describe analogous results assuming vaccination and transgenic population reduction are combined with an antimicrobial medication strategy that removes infectious hosts at the same rate as the background recovery rate, while (E-F) describe the results when an antimicrobial medication strategy that removes infectious hosts at twice the background recovery rate is used. Panels (A-B) illustrate how absent transgenic manipulation long-term pathogen elimination is only possible in our model if the fraction of hosts vaccinated exceeds approximately 0.78, which corresponds to roughly 1 − 1/*T*_*R*_ (e.g., [85]) for the parameter values in Table 1.

Combining the different strategies can have a distinctly non-additive effect on reducing long-term incidence. Fig 3 presents the effects of combining clinical interventions with transgenic vector manipulation strategies that aim to reduce vector population recruitment. We highlight that the medication-induced recovery rate can govern how sensitive the type reproduction number at the new endemic equilibrium is to reductions in vector recruitment when more than half the hosts are effectively vaccinated. At faster medication-induced recovery rates, raising the vaccination fraction or reducing vector recruitment have increasingly similar, qualitative effects on facilitating eventual pathogen elimination. Very similar conclusions hold when transgenic population replacement fails to drive the relative vector competence to zero (S4 Text).

**Fig 4:**
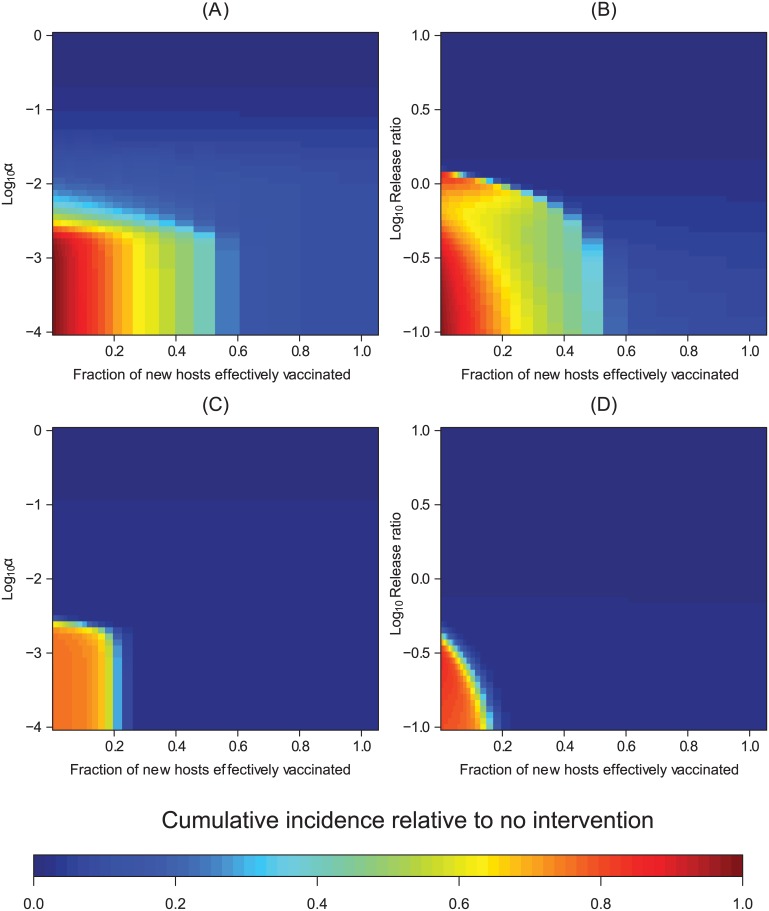
Epidemiological effects of integrating clinical interventions with transgenic manipulation over a 10-year time horizon. All panels on the left-hand side depict results for transgenic vector manipulation that aims at population replacement, while panels on the right-hand side depict results for transgenic vector manipulation aiming at population reduction. All panels describe the total number of cases (cumulative incidence) over the time horizon relative to the same quantity in the absence of any public health management programs ($\hat{q_{P}}\left(T_{H}\right)$; see Eq (5).) Panels (A-B) show the effect on $\hat{q_{P}}\left(T_{H}\right)$ when no antimicrobial medication is administered in addition to a vaccination program. In (C-D), the medication-induced recovery rate is 1/30 per day, which is 20% of the natural recovery rate.

The results above pertain to long-term dynamics. The rate at which transgenic manipulation changes vector populations can also affect the epidemiology of the vector-borne disease during a shorter, 10-year time horizon (Fig 4). Nevertheless, somewhat different dynamics can emerge between transgenic population reduction and transgenic population replacement. In particular, if successful transgenic population replacement monotonically reduces vector competence, long-term disease suppression results when the vaccination fraction is small (Fig 4A). By contrast, transgenic population reduction using relatively low release ratios (e.g., of about 1:1 in our model) need not lead to population elimination. When neither vaccines nor antimicrobial drugs have a strong impact, the persistence of the vector in the system allows for transient increases in infections following the end of the honeymoon period (Fig 4B), even when long-term incidence can eventually be reduced below the disease-endemic equilibrium (e.g., Fig 3). Finally, we note that under both transgenic population replacement and population reduction, even when the medication-induced recovery rate is low, it can compensate for slow transgenic manipulation or a low vaccination fraction in reducing the total number of cases over a 10-year time horizon (Fig 4C and 4D).

### Combining unsustainable transgenic population manipulation with partially effective clinical interventions

**Fig 5:**
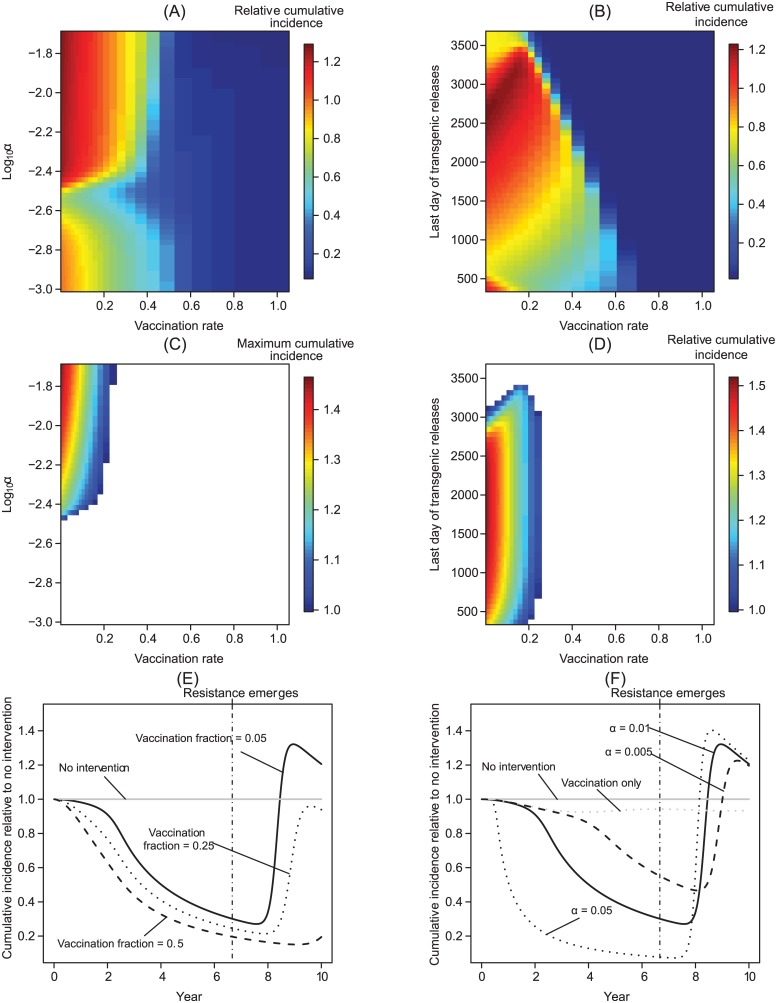
Unsustainable transgenic interventions. The epidemiological effects of integrating a vaccine-based intervention strategy with transgenic vector manipulation over a 10-year time horizon when the effects of the transgenic strategy are unsustainable. Here, and in Fig 6, resistance arises (or, in the case of population reduction, transgenic releases end) two thirds of the way into a transgenic manipulation regime unless noted otherwise. For the case of population replacement, we model initial invasion of the resistant pathogen by having a single vector carrying the mutant resistant pathogen appear at *t* = *τ*_*R*_; in this, and in subsequent figures, the parameter values other than *G*(*t*) are modeled to be the same for vectors and hosts infected with the resistant pathogen. (A) The total number of cases (cumulative incidence) relative to the total number of cases without any public health management program when transgenic vector manipulation aims at population replacement. (B) The total number of cases relative to the total number of cases without any public health management program when transgenic vector manipulation aims at reducing vector recruitment. (C-D) The maximum total number of cases, relative to the same quantity without intervention, when the vaccination fraction is low (i.e., $\hat{q_{P}}\left(\cdot \right)$ evaluated at the time point *T*_*H*_ where $\hat{q_{P}}\left(T_{H}\right)$ is maximized). In contrast to other figures, panels (C) and (D) illustrate the total number of cases at a given point in time rather than the total number of cases over the 10-year time horizon, with the white region of the plots corresponding to regions where the total number of cases with the intervention is always below the incidence in the absence of an intervention. We note that when a relatively small fraction of new hosts are vaccinated, abruptly ending a population reduction program can cause transient oscillations, leading to the nonlinearities apparent in panels (B,D). Panels (E-F) illustrate how the total number of cases can temporarily increase relative to no intervention if transgenic population replacement is unsustainable, although over much longer time horizons $\hat{q_{P}}\left(T_{H}\right)$ eventually falls below one. Panel (E) shows how vaccination can maintain a lower total number of cases relative to the situation in the absence of interventions, but still results in an increase in the total number of cases as vector competence recovers. In panel (F), 5% of new hosts are effectively vaccinated, but the decline *G*(*t*) in vector competence is not sustainable.

Transgenic vector manipulation strategies that fail to cause long-term changes to vector competence or recruitment require a high vaccination fraction to reduce the total number of cases over a 10-year time horizon to near zero levels, except when vector population extinction results (Fig 5A and 5B). When the vaccination fraction is low, herd immunity can be lost during the period when vector competence or population size is reduced. As vector competence recovers, this can temporarily increase the number of infectious hosts when a failed transgenic vector manipulation is carried out in conjunction with an intervention with a low vaccination fraction (Fig 5C–5F). Thus, a higher vaccination fraction is required to prevent increasing the total number of cases above levels predicted in the absence of control strategies.

**Fig 6:**
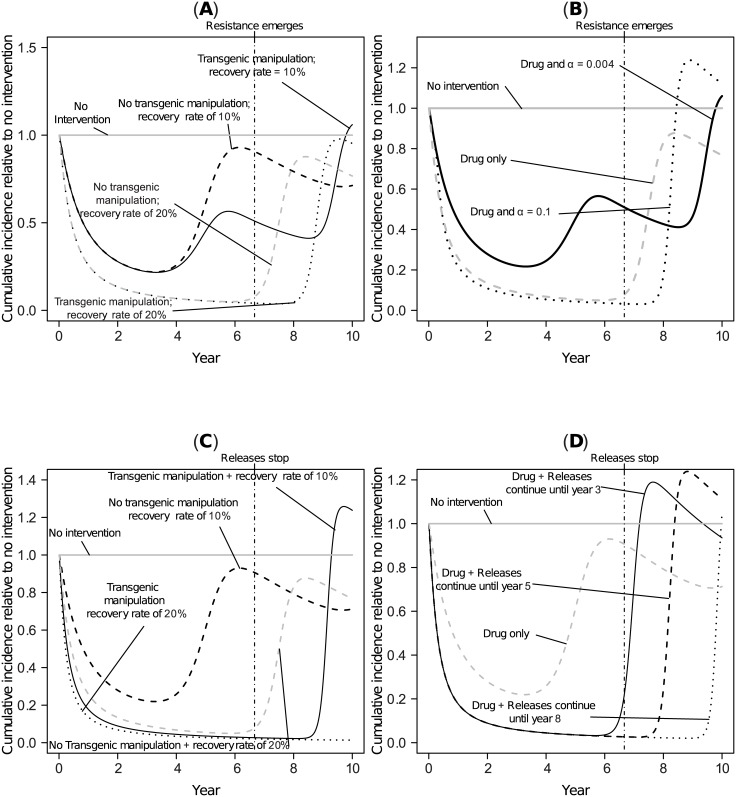
Transient effects on incidence of combining transgenic vector manipulation with antimicrobial medications of varying efficacies. (A-B) results for transgenic manipulation aimed at population replacement. (C-D) The same analysis for the case when transgenic manipulation aims at population reduction. Panel (A) assumes *α* = 10^−2^; panel (B) assumes *ρ* = (*U*^⋆^ + *V*^⋆^). Panels (B, D) assume an additional medication-induced recovery rate that is 10% of the natural recovery rate for all simulations, but vary *α* (B) or the time *τ*_*m*_ until releases stop (D). The vertical dotted-dashed line represents the point in time where resistance arises or releases end.

Transient increases in the total number of cases over a 10 year period can also result when medications are administered in an attempt to compensate for failed transgenic manipulation. In particular, a strategy combining antiviral medications with only transiently effective transgenic vector manipulation can result in larger transient oscillations than can arise when only the antimicrobial medication is administered (Fig 6). When the drug-induced recovery rate is much lower than the natural recovery rate, even an intervention based on antimicrobials can fail to prevent a transient increase in incidence following the recovery of vector competence or recruitment, although a marginally more effective antimicrobial intervention may still reduce the total number of cases below the level predicted in the absence of any intervention.

## Discussion

Although much research effort focuses on developing vaccines or medications for many vector-borne diseases (e.g., [13, 14, 86, 87]), and several transgenic vector manipulation strategies have been proposed ([16]), using either approach alone has yet to prove sufficient for managing many vector-borne diseases in the field (e.g., [16, 75, 88]). A comparative analysis is therefore key to elucidating how the interplay between clinical interventions and transgenic vector manipulation can minimize incidence and prevent pathogen endemicity.

Using parameter values for a single dengue serotype as a case study, we illustrate that when combined with a partially effective vaccine, accelerating the decrease in vector competence or recruitment can facilitate reductions in incidence over a moderate time horizon (between several years and a decade) provided such reductions can be maintained. However, fluctuations in incidence that result from transgenic manipulations that only temporarily lower recruitment or competence can be amplified when modestly effective clinical interventions are used following the end of the “honeymoon period”. This occurs as susceptible hosts accumulate over relatively longer demographic time scales, illustrating how differences between the time scale of the intervention and the time scale of host population dynamics can alter transient dynamics when intervention strategies are of limited efficacy.

We note that the adverse epidemiological consequences of only transiently effective interventions are likely not unique to transgenic manipulation strategies. Similar dynamics have also been commented upon for a model by [89] exploring the epidemiological effects of pulse vaccination strategies. In their model, incidence increased as vaccination pulses became more frequent, in part due to the inflow of susceptibles into the population. Similarly, in models analyzing the consequences of varying dengue vaccination coverage in seasonal environments in the presence of ongoing source removal, [33] found that poor vaccine coverage could actually cause transient increases in prevalence (although it is not clear from the results in [33] whether cumulative incidence also increases relative to the case without vaccination in their model). In principle, the evolution of antimicrobial resistance by pathogens may also exhibit similar adverse effects on incidence ([90]). Thus, a long time horizon accounting for host demographic shifts may be necessary to discern the epidemiological consequences of alternative intervention strategies, at least for systems beginning at a disease-endemic equilibrium (see also [83, 91]). In light of our analyses, cluster randomized trials assessing the efficacy of transgenic mosquito releases ([92]) or vaccines ([93]) should be sensitive to the potential for delayed feedback from intervention strategies to epidemiological dynamics, particularly in systems starting at a disease-endemic equilibrium ([89]). Our results also potentially highlight the risks of prematurely abandoning a transgenic manipulation strategy (or any other public health intervention), although in practice community support and resource allocation may wane over much shorter time scales than those considered here (e.g., [30, 94, 95]). Transgenic manipulation aiming at population reduction in particular may prove costly to sustain indefinitely ([96]). When complete local eradication is not achieved, or vectors immigrate from other communities, vector population sizes may recover rapidly ([97, 98]). Our results show that failed population replacement can also entail adverse epidemiological effects. Nevertheless, strategies aimed at population replacement may be less prone to long-term failure than transgenic population reduction (for instance, by proving more robust to immigration by wild-type vectors—e.g, [79]). The conclusions we present highlight non-trivial epidemiological risks with adopting transgenic manipulation strategies that can be difficult to sustain.

While we conduct numerical analyses of the reduction in incidence, our primary objective here is to develop a heuristic appreciation for how combining alternative strategies can facilitate disease management objectives. Thus, we seek to enhance our intuition for the nature of epidemiological effects a combined strategy can have, rather than make precise, quantitative predictions about any particular system or transgenic strategy. For the case of transgenic population replacement, our analyses focus on the functional form of the trajectory of the decline in vector competence, rather than the exact change in this quantity predicted by a model of any one transgenic population replacement strategy. This allows us to abstract the details of how transgenic manipulation changes the genetic profile of a vector population, and focus instead on their impact on the epidemiological dynamics of the manipulation. While we sacrifice some accuracy, results based on our logistic approximation have the benefit of being expected to apply, at least to a first approximation, across a range of proposed transgenic population replacement strategies (expectations that are supported—at least for the case of *Wolbachia*—by our analysis in S3 Text).

Our model enables us to characterize a baseline set of expectations from which analyses incorporating system-specific complications, such as seasonality, spatial structure, and pathogen diversity (e.g., multiple serotypes) can build. Model (1) thereby provides a potential point of departure should statistical comparisons to epidemiological data in specific systems show the model to be unable to capture observed dynamical behavior in the absence of clinical interventions. For instance, multiple serotypes can be modeled by the addition of new compartments characterizing alternative infectious states for hosts and vectors to our model. Given the importance of acquired immunity (either following infection or vaccination) to the dynamics we report, we also highlight the need to extend our framework to vector-borne diseases with limited acquired host immunity following infection (such as malaria and trypanosomiasis) as an important direction for future research. Seasonality presents another potential complication. Model (1) may be able to account for its effects by introducing a time-varying delay in the transition of hosts from the susceptible to infectious compartments to represent temporal variability in the extrinsic incubation period. Seasonal fluctuations could also drive vector population dynamics (e.g., [99]); one way in which such influences could be modeled is by multiplying the vector’s unconstrained recruitment rate *F*(*U*, *V*) by a periodic function describing, for instance, intra-annual variability in rainfall or temperature. Finally, alternative vector species can potentially replace the primary vector following transgenic population reduction (e.g., [100]). Being species-specific, transgenic population reduction may be more prone than traditional, less-species specific control measures such as insecticide spraying to allowing susceptible hosts to accumulate as a novel vector emerges. Modifying model (1) to include a (potentially less competent) competitor vector ([101]) may allow comparing the relative advantages of integrating clinical interventions with either less discriminatory control strategies or with highly targeted transgenic reduction. This work thus provides a framework for ascertaining how these and other sources of heterogeneity may interact with integrated management strategies.

For several vector-borne diseases, both transgenic manipulation and trial vaccination programs have come under some criticism for being only partially effective or practical (e.g., [13, 102] and [16]). Integrating clinical and transgenic manipulation strategies may therefore be an attractive and even currently feasible proposition. We find that when transgenic manipulations are unsustainable, combining ineffective strategies can, at least over the short term, result in higher incidence than would have been predicted in the absence of an intervention. Still, our results also indicate that public-health objectives could be attained even with partially effective strategies, provided suitable combinations of both approaches can be adopted.

## Supporting Information

S1 Text Equilibria for the host-vector-pathogen system in the main text in the absence of control. (PDF)

S2 Text Approximating explicit population-genetic models for the spread of a transgenic, anti-pathogen construct using a phenomenological description *G*(*t*) of change in mean vector competence. (PDF)

S3 Text Comparing resultant epidemiological dynamics using a phenomenological description of the decline *G*(*t*) in vector competence against dynamics predicted by an explicit model of the spread of an anti-pathogen construct (*Wolbachia*). (PDF)

S4 Text The long-term effect of integrating transgenic population replacement with clinical interventions when relative vector competence cannot be lowered beyond a fixed threshold. (PDF)

S5 Text R scripts for numerical simulations. (R)
