# Supplementary material for: Integrating Transgenic Vector Manipulation with Clinical Interventions to Manage Vector-Borne Diseases
Source: PLoS Comput Biol. 2016 Mar 10;12(3):e1004695. doi: 10.1371/journal.pcbi.1004695 (PMC4786096; doi:10.1371/journal.pcbi.1004695)
Supplement: S1 Text — (PDF) [file pcbi.1004695.s001.pdf]

Supplementary Information S1 - Equilibria for the host-vector-pathogen system in the main text in the absence of control

In the absence of control programs, there are several equilibria for system (1) in the main text. After some algebra, it can be shown that the equilibrium density $S^\star$ of susceptibles is given by $\frac{1}{2ad^2k(a\mu-d\phi)}(\pm k\sqrt{d^2\left(\frac{(kS_M(2a\mu(d+g)+ad\phi-d\phi(d+g))+d\psi\phi(\mu-r)(a+d+g))^2}{k^2}-4aS_M^2(d+g)(a\mu-d\phi)(d(\mu+\phi)+g\mu)\right)}+$ $d^2(kS_M(g\phi-a(2\mu+\phi))+\psi\phi(a+g)(r-\mu))-2adgk\mu S_M+d^3\phi(kS_M+\psi(r-\mu)))$

The equilibrium number of recovered hosts $R^\star = \frac{\sqrt{\phi}\sqrt{S^\star(4\mu\psi(r-\mu)(a+d+g)+d^2k\phi S^\star-2dkS_M\phi)+kS_M^2\phi}+\sqrt{k}(S^\star(d\phi-2\mu(a+g))-S_M(2\mu+\phi))}{2\sqrt{k}\mu(a+d+g)}$, where $S^\star$ is given by the corresponding equilibrium density of susceptible hosts.

For each $S^\star$, the remaining equilibrium densities are given by $I^\star = \frac{S_M-dS^\star}{a+d+g}$, $V^\star = \frac{(r-\mu)\phi I^\star}{k((\mu+\phi)I^\star+\mu(R^\star+S^\star))}$ and $U^\star = \frac{(r-\mu)\mu(I^\star+R^\star+S^\star)}{k((\mu+\phi)I^\star+\mu(R^\star+S^\star)}$.

Two other important equilibria are the vector-free equilibrium, where the equilibrium density of susceptible hosts $S^\star = S_M/d$, and the pathogen-free equilibrium with the vector present, where the equilibrium susceptible host density is the same as the vector-free equilibrium and the equilibrium vector density $U^\star = \frac{r-\mu}{k}$.

There are four unique eigenvalues of the Jacobian matrix for system (1) in the main text evaluated at the pathogen-free equilibrium: $e_1 = -d$, $e_2 = -\mu-(r-2\mu)m(t)$, $e_3 = -\frac{\sqrt{kS_M(kS_M(a+d+\delta+g-\mu)^2+4d\psi(r-\mu)\phi(t))}+kS_M(a+d+\delta+g+\mu)}{2kS_M}$, and $e_4 = \frac{\sqrt{kS_M(kS_M(a+d+\delta+g-\mu)^2+4d\psi(r-\mu)\phi(t))}-kS_M(a+d+\delta+g+\mu)}{2kS_M}$. When the vector populations are viable at the pathogen-free equilibrium (i.e., $rm(t) > \mu$), e_4 is the dominant eigenvalue, and when $0 \le \epsilon \le 1$, after some algebra it can be shown that $e_4 < 0$ whenever condition (3) in the main text is satisfied.
