# Supplementary material for: Integrating Transgenic Vector Manipulation with Clinical Interventions to Manage Vector-Borne Diseases
Source: PLoS Comput Biol. 2016 Mar 10;12(3):e1004695. doi: 10.1371/journal.pcbi.1004695 (PMC4786096; doi:10.1371/journal.pcbi.1004695)
Supplement: S2 Text — (PDF) [file pcbi.1004695.s002.pdf]

Supplementary Information S2: Approximating explicit population-genetic models for the spread of a transgenic, anti-pathogen construct using a phenomenological description $G(t)$ of change in mean vector comptence

System (1) in the main text represents the effects of transgenic population manipulation on average vector competence, and describes how these manipulations affect prevailing epidemiological dynamics. We model situations where there is little or no feedback from on-going epidemiological dynamics on the implementation of a transgenic release program aimed at population replacement. This is reasonable when, for instance, the release regimes for transgenic vectors or the specific transgenic construct follow a fixed release ratio over the relevant time horizon. Indeed, if transgenic constructs work as expected and release regimes are constant, prevailing vector population and epidemiological dynamics will not affect the change in vector comptence.

When vector fitness is unaffected by pathogen load (see refs. [6,9] of the main text for exceptions), transgenic manipulation aimed at population replacement should change the average phenotype of vectors, but is unlikely to change vector population dynamics. This is because most release programs envision releasing either non-vectoring transgenic males (whereas reproductive output of a vector population often depends on female density - e.g., [1, 2]), or because strong, density-dependent recruitment can quickly restore vector population sizes to equilibrium even when transgenic females are released (e.g., [3]).

Indeed, describing the change in average vector competence therefore only requires tracking the underlying gene frequency of an anti-pathogen transgene in the vector population. Below, we use explicit population genetic models to demonstrate how the spread of a wide array of proposed transgenic constructs can be closely approximated by the function $G(t) = 1/[1 + \exp(\alpha t + \beta)]$.

Case 1. The spread of an anti-pathogen transgene in the absence of gene drive

It is commonly assumed that introgressing anti-pathogen constructs into wild vector populations requires linking anti-pathogen genes to a selfish genetic element (SGE - i.e., genes that are inherited at higher rates than other genes in the genome). This is because the release numbers required to render vector populations vector incompetent are asssumed to be prohibitive without such techniques (e.g., [4], ref. [16] of the main text). However, some recent theoretical studies suggest that transgenic population replacement may be feasible absent gene drive ([3], ref. [9] of the main text), and these results appear robust using considerably more detailed, mechanistic models of urban mosquito populations (ref. [79] of the main text).

If vector population dynamics follow a logistic growth model, and the transgenic construct does not affect the density-independent survivorship of adult vectors (e.g., ref. [79] of the main text), then the dynamics of all vectors (n) and vectors carrying an anti-pathogen gene unlinked to a SGE (n_A) are given by

$$\begin{aligned} \frac{dn}{dt} &= n(w_{AA}(\frac{n_A}{n})^2 + 2w_{Aa}(\frac{n_A}{n})(1-\frac{n_A}{n}) + w_{aa}(1-\frac{n_A}{n})^2)(1-n/K) - \mu n \qquad (1) \\ \frac{dn_A}{dt} &= n(w_{AA}(\frac{n_A}{n})^2 + w_{Aa}(\frac{n_A}{n})(1-\frac{n_A}{n}))(1-n/K) - \mu n_A \end{aligned}$$

(e.g., [5]). Here, A, a represent the presence or absence of the transgenic construct, $w_{i,j}$ represents the per-capita contribution of individuals with genotype i, j to recruitment, n_A describes the number of gametes of type A, K describes the carrying capacity and μ describes the per-capita, density-independent adult vector mortality.

There are three noteworthy consequences of model (1). First, the release of transgenic vectors can be modeled as increasing the relative contribution to recruitment by vectors homozygous for the anti-pathogen construct. This is true, for instance, when containers with transgenic mosquito eggs are released into the vector's habitat (e.g., refs. [79, 97, 98] of the main text). We note that under such a release regime, if the containers are manufactured to prevent oviposition (e.g., ref. [97] of the main text), the carrying capacity in model (1) of the

main text remains unaffected. Thus, system (1) can describe the release of transgenic vectors for the duration of the time horizon of interest, and models the spread of an anti-pathogen construct in the absence of gene-drive.

Second, the dynamics described by system (1) can also result when carrying the transgene confers a net fitness advantage to individual vectors (e.g., ref. [6] of the main text). This scenario could arise if the transgene also manages to confer resistance to other pathogens of the vector, even if the focal pathogen that is the target for elimination (and hence subject to an epidemiological feedback) does not detrimentally affect the vector. How common such a scenario is depends on the particular microbiome of different vector populations; we simply note here that equations (1) can potentially describe such a scenario.

Finally, system (1) can be used to describe not only the introgression of an anti-pathogen gene, but how an anti-pathogen gene carrying a fitness cost can be lost from the vector population. In particular, sustained releases might temporariy compensate for such a fitness cost so that $w_{AA} > w_{aa}$. However, if releases cease, then the fitness cost of the transgene may become apparent, rendering $w_{aa} > w_{Aa}, w_{AA}$. Reinterpreting n_A as the number of vectors not carrying the transgene can allow us to model how a transgene with a fitness cost can be lost from the vector population.

The dynamics of the transgene's frequency can then be calculated by numerically solving system (1). Figure (A) shows how a transgene can increase in frequency according to the dynamics predicted by integrating system (1). When the transgene's expression is dominant (so that carrying a single copy suffices to render an individual vector incompetent - e.g., [6]), the relative average magnitude $G(t)$ of vector competence in the population is completely described by one minus the frequency of the anti-pathogen construct. As is apparent from Figure (A), the decline in vector-competence can be approximated very closely by a sigmoidal function of the form $G(t) = 1/[1 + \exp(\alpha t + \beta)]$. We note that, by symmetry, this functional form also describes the loss of an anti-pathogen transgene (or an increase of the wildtype genotype) that carries a noticeable fitness cost following the end of releases.

Case 2. The spread of *Wolbachia*

Mosquitoes infected with the symbiotic insect bacteria *Wolbachia* (e.g., [7]) have been shown to be unable to vector several medically important pathogens including malaria, chikungunya and dengue (ref. [56] of the main text). *Wolbachia* is vertically transmitted, and the symbiont spreads by manipulating host reproduction to favor the reproductive contribution of infected females. In particular, cytoplasmic incompatibility (CI), where only infected females are able to carry the offspring of infected males, is an important mechanism facilitating *Wolbachia* spread.

Following [8], the dynamics for the frequency $x(t)$ of a single *Wolbachia* strain for a vector population at equilibrium are given by

$$\frac{dx}{dt} = rQ(1-\tau x)x^2 - rx(1-\tau) \tag{2}$$

where r is the per-capita recruitment rate, τ is the fraction of infected offspring that carry *Wolbachia* (because *Wolbachia* transmission from mother to offspring may be imperfect), Q describes the magnitude of cytoplasmic incompatibility, and because we consider cases where transgenic population replacement does not alter vector population dynamics, *Wolbachia* infected individuals are assumed to suffer no further density-dependent mortality effects (which is reasonable in *Ae. aegypti* - e.g., ref. [11] of the main text).

When *Wolbachia* infection prevents vectors from transmitting the pathogen, the population-wide average *Wolbachia* infection rate directly describes average vector-competence. Again, as is apparent in Figure (B), the decline in vector-competence caused by releasing *Wolbachia* can be closely approximated by a sigmoidal function of the form $G(t) = 1/[1 + \exp(\alpha t + \beta)]$.

Case 3. The spread of a MEDEA-linked transgenic construct

Non-Mendelian inheritance of MEDEA and MEDEA-like constructs results from mothers carryng the construct being unable to produce offspring that fail to inherit a copy of the construct ([9]). The severity of such maternal effects on offspring fitness can be described by a parameter T that determines the probability of a wildtype homozygote offspring being killed by a heterozygotic mother.

If the vector population is at stable equilibrium and the vector population size is sufficiently large (so that the effects of genetic drift can be neglected), the dynamics of MEDEA or a MEDEA-like construct and the wildtype gene among female vectors between generations $t, t+1$ is given by

$$\begin{aligned} D_{AA}(t+1) &= pS(pD - s(D_{AA}(t) + hD_{Aa}(t)/2))/W \quad (3) \\ D_{Aa}(t+1) &= ((1-pS)(pD - s(D_{AA}(t) + hD_{Aa}(t)/2)) + pS(1 - pD - shD_{Aa}(t)/2))/W \\ D_{aa}(t+1) &= ((1-pS)(1 - pD - (sh + T - Tsh)D_{A,a}(t)/2))/W \end{aligned}$$

([10]). Here, $D_{i,j}(t)$ represents the frequency of individuals with genotype i, j on generation t, $pS = pD$ is the fraction of males and females carrying a MEDEA or MEDEA-like construct (A), s is the fitness cost or benefit to carriers relative to wildtype (a) homozygotes, and h describes the dominance effect of MEDEA on fecundity. The composite parameter W describes mean fitness and is given by $W = (1 - s(D_{AA} + hD_{Aa}/2) - shD_{Aa}/2 - D_{Aa}/2(1-sh)(1-pS))$.

Assuming the transgene with dominant expression is linked to the MEDEA construct, Figure (C) shows that despite system (3) being framed as a discrete-time model, the decline in vector-competence can be approximated by a continuous function over a sufficiently long time horizon. The functional form $G(t) = 1/[1 + \exp(\alpha t + \beta)]$ once again describes well the basic dynamics of the reduction in vector competence resulting from MEDEA spread.

Conclusions

In model (1) of the main text, transgenic population replacement does not involve a feedback from epidemiological dynamics to the trajectory of vector competence. Thus, we are able to model how transgenic manipulation affects epidemiological dynamics using a function $G(t)$ that does not depend on other state variables in our model. Here we show that the phenomenological function $G(t) = 1/[1+\exp(\alpha t+\beta)]$ is able to closely capture the dynamics of several transgenic strategies that vary considerably in their underlying biology. Thus, our approach captures the basic dynamics of transgenic population replacement despite considerable differences in the biological details underlying any given transgenic strategy.

The close congruence between the trajectories predicted by the models incorporating explicit population genetics (Figs. A-C) implies that using a generic, phenomenological description of transgenic manipulation in the form of $G(t)$ allows us to gain analytic tractability, and, perhaps more importantly, generate broader insights that can be expected to be robust across a range of specific transgenic approaches.

Substantially more detailed models incorporating greater biological realism would be appropriate for evaluating the epidemiological effects of combining clinical interventions with transgenic manipulation for specific vector species, localities, and diseases. They could also help pinpoint the limitations of specific transgenic strategies when combined with other public health strategies in each system. Nevertheless, we argue that these are not the only useful goals of modeling. A general understanding of the basic principles of the interplay between transgenic manipulation, clinical interventions and epidemiological dynamics, which can be expected to hold across a range of transgenic strategies, can be of value in framing and guiding the analysis, as well as interpreting the results, of considerably more detailed, system-specific models. To attain such a baseline understanding, a phenomenological approach which closely describes the dynamics of a range of transgenic manipulations can often suffice.

References

[1] Sota T, Mogi M (1989) Effectiveness of zooprophylaxis in malaria control: a theoretical inquiry, with a model for mosquito populations with two bloodmeal hosts. Medical and Veterinary Entomology 3: 337–345.

[2] Focks DA, Haile DG, Daniels E, Mount GA (1993) Dynamic life table model for *Aedes aegypti* (Diptera: Culicidae): analysis of the literature and model development. Journal of Medical Entomology 30: 1003–1017.

[3] Robert M, Okamoto KW, Gould F, Lloyd AL (2014) Anti-pathogen genes and the replacement of disease-vectoring mosquito populations: a model-based evaluation. Evolutionary Applications 7: 1238–1251.

[4] Sinkins SP, Gould F (2006) Gene drive systems for insect disease vectors. Nature Reviews Genetics 7: 427–435.

[5] Hasofer AM (1966) A continuous-time model in population genetics. Journal of Theoretical Biology1 11: 150–163.

[6] Huang Y, Magori K, Lloyd AL, Gould F (2007) Introducing transgenes into insect populations using combined gene-drive strategies: modeling and analysis. Insect Biochemistry and Molecular Biology 37: 1054–1063.

[7] Caspari E, Watson GS (1959) On the evolutionary importance of cytoplasmic sterility in mosquitoes. Evolution 13: 568-570.

[8] Keeling MJ, Jiggins FM, Read JM (2003) The invasion and coexistence of competing Wolbachia strains. Heredity 91: 382–388.

[9] Beeman RW, Friesen KS, Denell RE (1992) Maternal-effect selfish genes in flour beetles. Science (New York, NY) 256: 89–92.

[10] Wade MJ, Beeman RW (1994) The population dynamics of maternal-effect selfish genes. Genetics 138: 1309–1314.

Figure captions

Figure A. The proportional decline in average vector competence caused by releasing transgenic vectors carrying constructs unlinked to gene-drive mechanisms. The decline in average vector competence (scaled to initial vector competence) is simply one minus the frequency of the antipathogen transgene in the vector population. The relative recruitment rates are modified by releasing different numbers of transgenic vectors, and are varied across panels. The remaining parameter values are obtained from Table 1 of the main text. In this, and in subsequent figures, the solid black line represents the prediction of the genetically explicit model, and the grey dots represents the phenomenological function $G(t)$ evaluated at time point t with parameter values that most closely approximate the trajectory predicted by the explicit genetic model. The parameters α, β represent the parameters of the sigmoidal function $G(t)$ fit by performing a nonlinear least squares regression of $G(t)$ on the solution to the genetically explicit model.

Figure B. The proportional decline in vector competence following the release of transgenic vectors infected with *Wolbachia* (solid black lines) and the phenomenological function $G(t)$ most closely approximating the subsequent spread of *Wolbachia* (dotted grey lines). The decline in average vector competence (scaled to initial vector competence) is simply one minus the frequency of *Wolbachia* infection in the vector population. The extent of cytoplasmic incompatibility (CI) is varied across panels. For *Ae. aegypti* infected by the *wMel*, the CI is typically near 1 (e.g., ref. [11] of the main text), but we consider a much wider range here to encompass both successful and failed transgenic population manipulation strategies. In all panels, initial *Wolbachia* prevelance is set at 2% and r is as in Table 1 of the main text.

Figure C. The proportional decline in vector competence caused by releasing transgenic vectors carrying a transgenic construct linked to a MEDEA-like element (solid black lines) and the phenomenological function $G(t)$ most closely approximating the subsequent spread of MEDEA (dotted grey lines). The decline in average vector competence (scaled to initial vector competence) is simply one minus the frequency of the MEDEA element in the vector population. The probability that an offspring without the MEDEA element is killed by a MEDEA-carrying mother is varied across panels. In all panels, initial MEDEA frequency is set at 1%, $s = 0$ and $h = 1$.

Figures

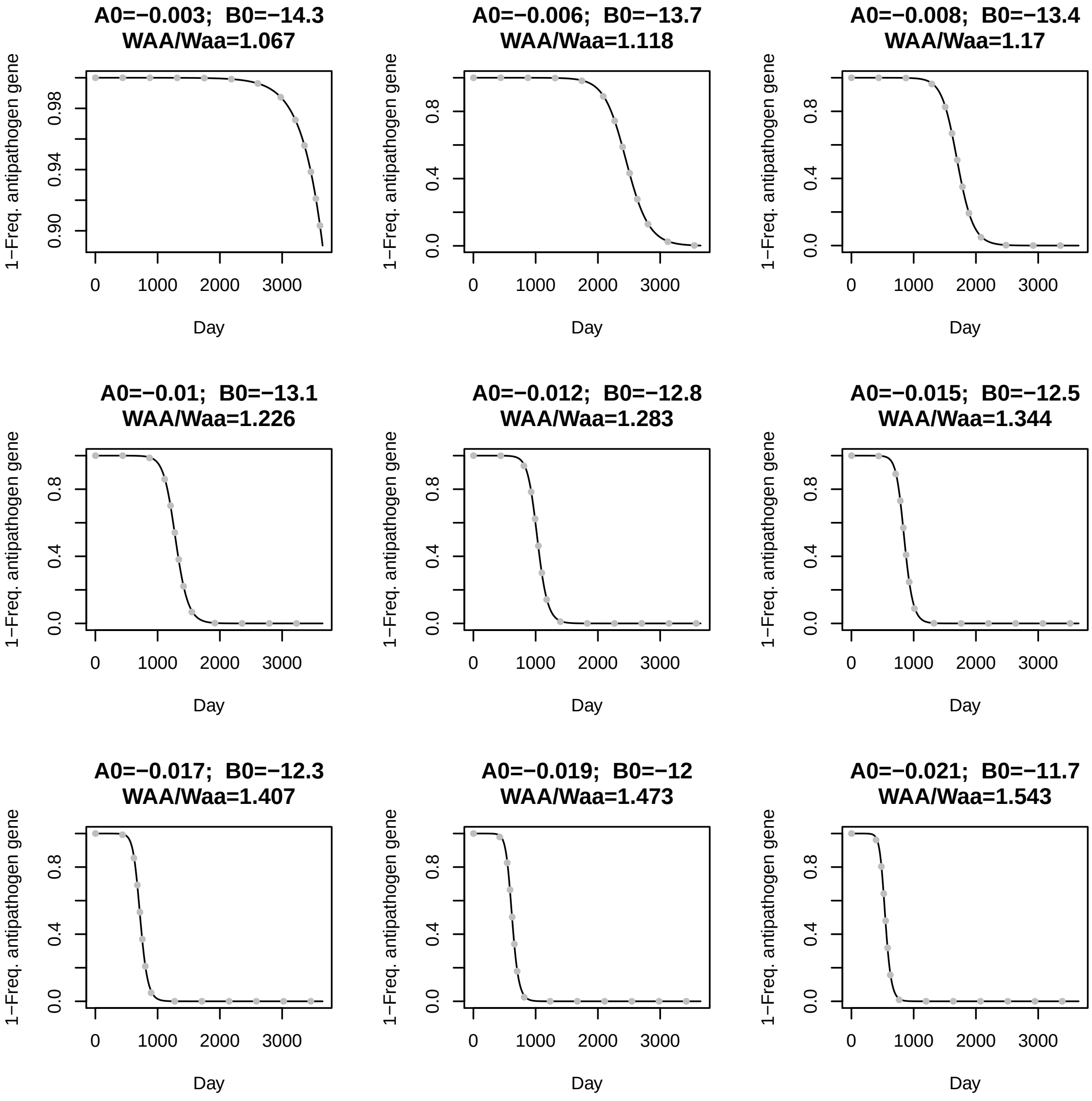
A0=−0.003; B0=−14.3
WAA/Waa=1.067
A0=−0.006; B0=−13.7
WAA/Waa=1.118
A0=−0.008; B0=−13.4
WAA/Waa=1.17
A0=−0.01; B0=−13.1
WAA/Waa=1.226
A0=−0.012; B0=−12.8
WAA/Waa=1.283
A0=−0.015; B0=−12.5
WAA/Waa=1.344
A0=−0.017; B0=−12.3
WAA/Waa=1.407
A0=−0.019; B0=−12
WAA/Waa=1.473
A0=−0.021; B0=−11.7
WAA/Waa=1.543
1−Freq. antipathogen gene
Day
0
1000
2000
3000
0.90
0.94
0.98
0.0
0.4
0.8

Figure A

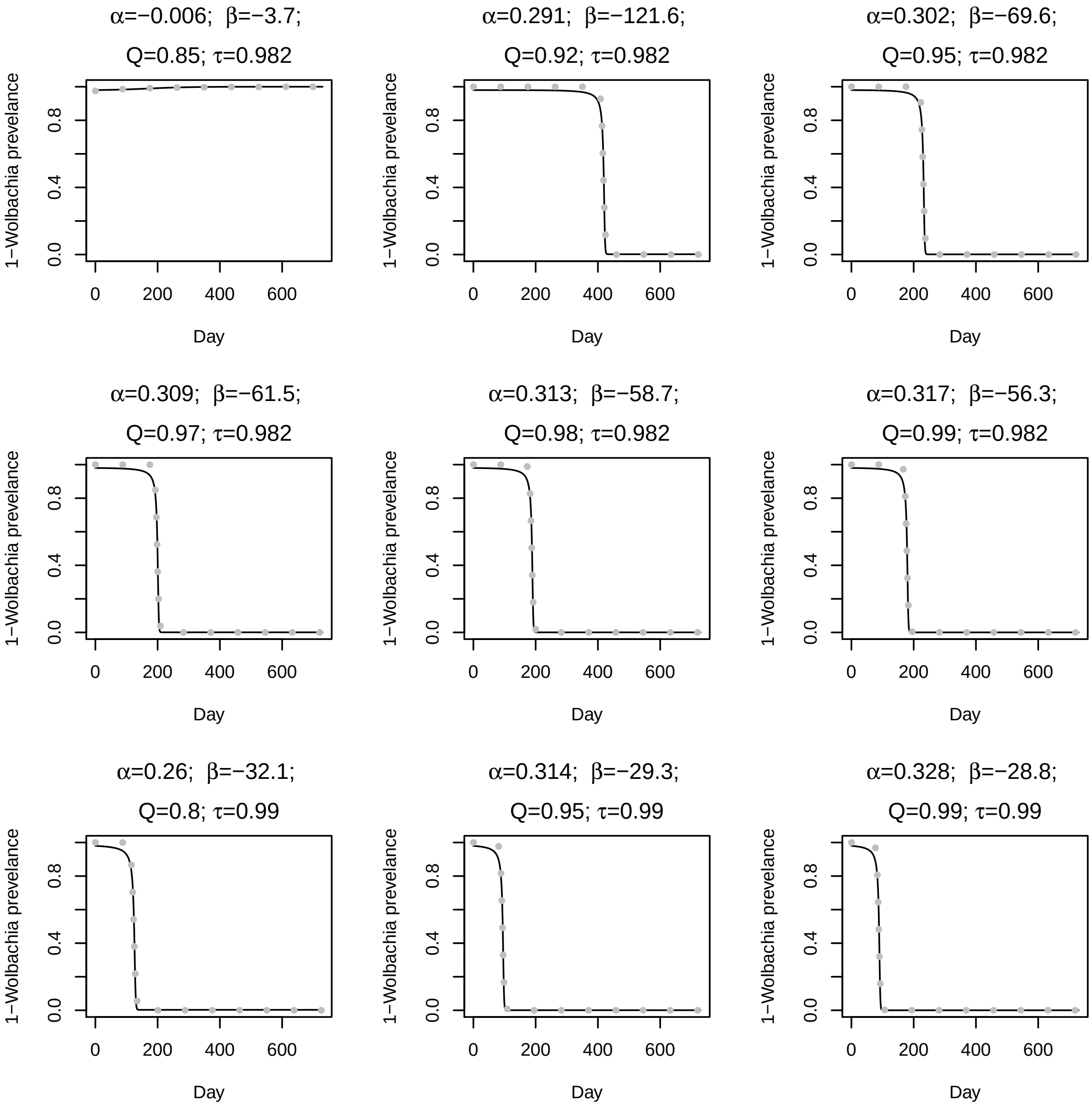
α=−0.006; β=−3.7;
Q=0.85; τ=0.982
α=0.291; β=−121.6;
Q=0.92; τ=0.982
α=0.302; β=−69.6;
Q=0.95; τ=0.982
α=0.309; β=−61.5;
Q=0.97; τ=0.982
α=0.313; β=−58.7;
Q=0.98; τ=0.982
α=0.317; β=−56.3;
Q=0.99; τ=0.982
α=0.26; β=−32.1;
Q=0.8; τ=0.99
α=0.314; β=−29.3;
Q=0.95; τ=0.99
α=0.328; β=−28.8;
Q=0.99; τ=0.99
1−Wolbachia prevelance
Day

Figure B

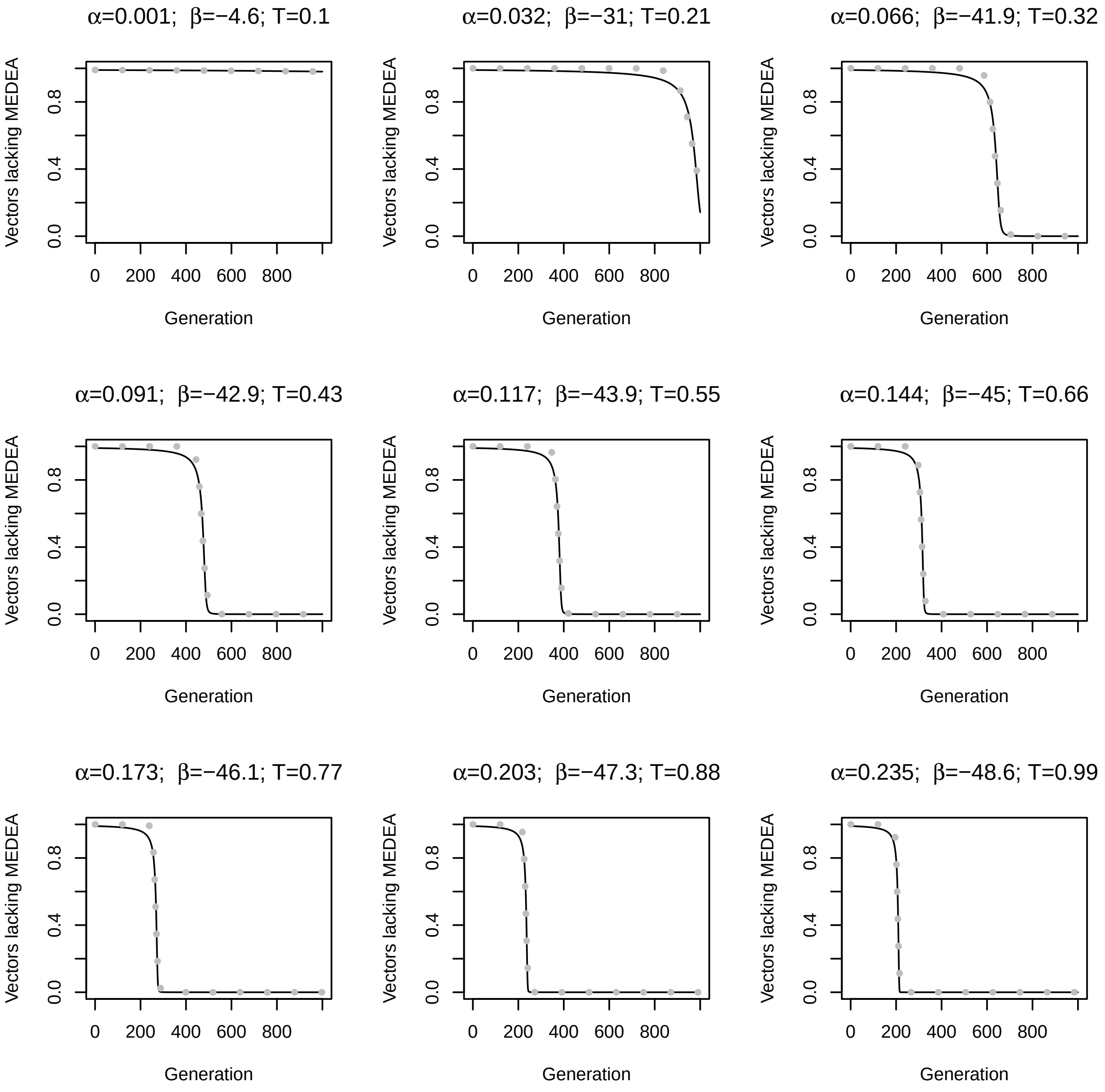

α=0.001; β=−4.6; T=0.1
α=0.032; β=−31; T=0.21
α=0.066; β=−41.9; T=0.32
α=0.091; β=−42.9; T=0.43
α=0.117; β=−43.9; T=0.55
α=0.144; β=−45; T=0.66
α=0.173; β=−46.1; T=0.77
α=0.203; β=−47.3; T=0.88
α=0.235; β=−48.6; T=0.99
Vectors lacking MEDEA
Generation
0.0
0.4
0.8
0
200
400
600
800

Figure C
