# Supplementary material for: Integrating Transgenic Vector Manipulation with Clinical Interventions to Manage Vector-Borne Diseases
Source: PLoS Comput Biol. 2016 Mar 10;12(3):e1004695. doi: 10.1371/journal.pcbi.1004695 (PMC4786096; doi:10.1371/journal.pcbi.1004695)
Supplement: S3 Text — (PDF) [file pcbi.1004695.s003.pdf]

Supplementary Information S3: Comparing resultant epidemiological dynamics using a phenomenological description of the decline $G(t)$ in vector competence against dynamics predicted by an explicit model of the spread of an anti-pathogen construct

We analyze the epidemiological dynamics for a variant of our model (1) in the main text in which the dynamics of the spread of an anti-pathogen construct (here, *Wolbachia*) in the vector population is modeled explicitly. Assuming that infection by the *Wolbachia* construct renders the individual vector completely refractory to the pathogen, the dynamics of the pathogen-vector-host-*Wolbachia* system can be described as

$$\begin{aligned}
\frac{dS}{dt} &= (1-\epsilon)b - \psi\frac{V_n S}{S+I+R} - dS \qquad \text{(S3-1)}\\
\frac{dI}{dt} &= \psi\frac{V_n S}{S+I+R} - (d+a+g+\delta)I\\
\frac{dR}{dt} &= \epsilon b + gI + \delta I - dR\\
\frac{dU_n}{dt} &= [r-k(U_n+V_n+U_w)](U_n+V_n+(1-\tau)U_w)(1-Q\frac{U_w}{U_n+V_n+U_w}) - \phi\frac{U_n I}{S+I+R} - \mu U_n\\
\frac{dV_n}{dt} &= \phi\frac{U_n I}{S+I+R} - \mu V_n\\
\frac{dU_w}{dt} &= \tau[r-k(U_n+V_n+U_w)](U_w) - \mu U_w.
\end{aligned}$$

Here, U_n describes the density of vectors neither carrying *Wolbachia* nor the pathogen, V_n describes the density of vectors that do not carry carry *Wolbachia* but are infectious for the pathogen, and U_w is the density of vectors that carry *Wolbachia*. The submodel combining vector population dynamics and the spread of *Wolbachia* is based on the model in [1], and the underlying assumptions lead to the *Wolbachia* frequency model used in Supplementary Information S2. As in Supplementary Information S2, τ describes the fidelity of transmission from mother to offspring and Q describes the probability of zygotic death due to cytoplasmic incompatibility. We follow [1] in describing a scenario where the male:female sex ratio is 1:1.

We compared the epidemiological dynamics predicted by model (S3-1) to those predicted by model (1) of the main text under a range of combined intervention strategies. For each

scenario, we numerically integrated model (S3-1) using the parameter values from Table 1 of the main text. We then fit the phenomenological function $G(t) = 1/(1 + \exp(\alpha t + \beta))$ to the predicted frequency of *Wolbachia* infected vectors using nonlinear least squares. We then numerically integrated model (1) in the main text using the fitted approximation function, and visually compared the dynamics for the total number of cases (the metric used in the main text) predicted by both models.

Figure A illustrates how in the absence of any clinical interventions (i.e., $\delta, \epsilon = 0$), both the phenomenological approximation $G(t)$ (solid black line) and the explicit model describing *Wolbachia* spread predict very similar dynamical patterns. The approximation generally does best when the intervention is either largely ineffective (low fidelity of vertical *Wolbachia* transmission) or highly effective (high fidelity of vertical *Wolbachia* transmission coupled with a high degree of cytoplasmic incompatibility). When the rate of spread of *Wolbachia* is at intermediate levels, the approximation $G(t)$ suggests somewhat higher cumulative incidence than would be predicted by model (S3-1).

We find that these conclusions appear robust when either a vaccine or antimicrobial drug is also deployed in conjunction with *Wolbachia* releases (Figs. B and C). Despite potential nonlinearities that could arise when the strategies interact following an only modestly effective clinical control measure (Fig. C), the epidemiological dynamics predicted by the phenomenological approximation and the model explicitly describing the spread of *Wolbachia* both show very similar trajectories.

To evaluate how the evolution of pathogen resistance to *Wolbachia* can influence subsequent dynamics, we integrated model (S3-1) until a time fixed time point τ_R, and from τ_R to the end of the time horizon integrated the following system of differential equations:

$$
\begin{aligned}
\frac{dS}{dt} &= (1-\epsilon)b - \psi\frac{(V_n+V_w)S}{S+I_n+I_w+R} - dS \qquad \text{(S3-2)}\\
\frac{dI_n}{dt} &= \psi\frac{(V_n)S}{S+I_n+I_w+R} - (d+a+g+\delta)I_n\\
\frac{dI_w}{dt} &= \psi\frac{(V_w)S}{S+I_n+I_w+R} - (d+a+g+\delta)I_w\\
\frac{dR}{dt} &= \epsilon b + g(I_n+I_w) + \delta(I_n+I_w) - dR
\end{aligned}
$$

$$
\begin{aligned}
\frac{dU_n}{dt} &= [r - k(U_n + V_n + U_w + V_w)](U_n + V_n + (1-\tau)(U_w + V_w))(1 - Q\frac{(U_w + V_w)}{U_n + V_n + U_w + V_w}) \\
&\quad -\phi\frac{U_n I_n}{S + I_n + I_w + R} - \mu U_n \\
\frac{dV_n}{dt} &= \phi\frac{U_n I_n}{S + I_n + I_w + R} - \mu V_n \\
\frac{dU_w}{dt} &= \tau[r - k(U_n + V_n + U_w)](U_w + V_w) - \phi\frac{U_w I_w}{S + I_n + I_w + R} - \mu U_w \\
\frac{dV_w}{dt} &= \phi\frac{U_w I_w}{S + I_n + I_w + R} - \mu V_w,
\end{aligned}
$$

where V_w is the density of vectors carrying *Wolbachia* which are also vector-competent for the resistant pathogen strain, and I_w is the density of hosts infected by the *Wolbachia* resistant strain. As in the analysis of model (S3-1) above, the phenomenological function $G(t)$ is fit using nonlinear least squares to the predicted *Wolbachia* frequency from time 0 to τ_R (which we fix as equal to 2/3 of T_H, as in the main text). We assume that $V_w, I_w = 0$ until $t > \tau_R$, when a single vector carrying the resistant pathogen strain invades the system.

The dynamics of the combined integration of models (S3-1) and (S3-2) are depicted in Figure D. In a few cases (e.g., second and fourth panel, top row) there are somewhat larger and transient quantitative differences than the case where population replacement remained stable for the duration of the time horizon. This occurs because following a prolonged build up of susceptibles, both model (1) of the main text and model (S3-2) predict a large increase in the type reproductive number $T_R(t)$ just prior to the reintroduction of a pathogen. Nonlinearities inherent in both models mean that small numerical differences between model (S3-2) and model (1) of the main text can, in some regions of parameter space, translate into transient, quantitative differences. We emphasize, however, that for both the explicit model of *Wolbachia* spread and the invasion of resistant pathogen predict very similar endpoints to the model based on our phenomenological approximation $G(t)$.

Figures (A-D) thereby demonstrate that the phenomenological model for the decline in vector competence readily reproduces the key dynamics predicted by the model that explicitly describes the spread of *Wolbachia*. In particular, the sharp spike in cumulative infections that exceeds the number of infectious predicted from no intervention, following the end of the "honeymoon period", is correctly captured in both models.

References

[1] Keeling MJ, Jiggins FM, Read JM (2003) The invasion and coexistence of competing *Wolbachia* strains. Heredity 91: 382–388.

Figures

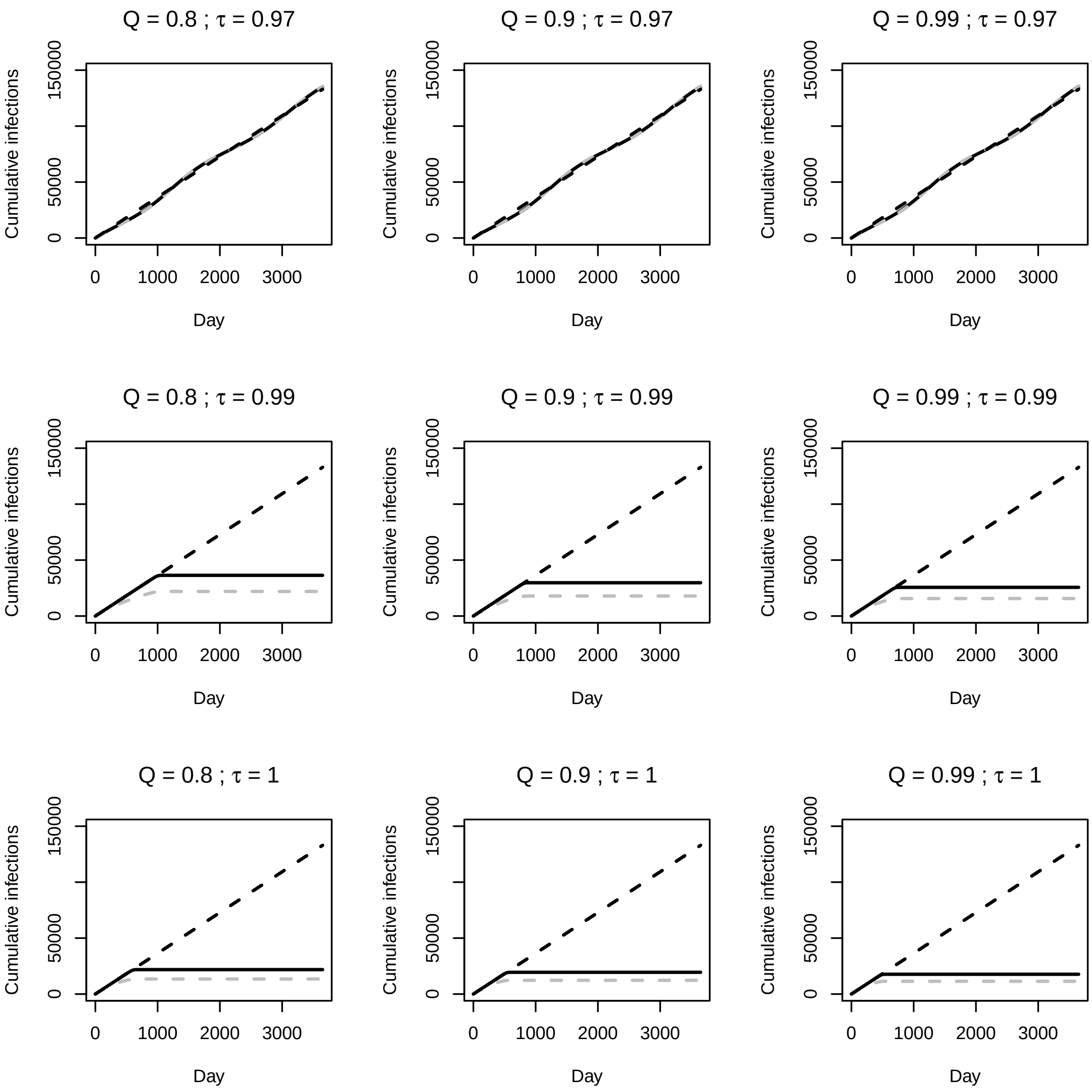

Figure A. The effects on cumulative incidence for model (S3-1) in the absence of clinical interventions. For *Ae. aegypti* infected by the *wMel*, both the CI and fidelity of vertical transmission are very high (near 1; e.g., ref. [11] of the main text), but we consider a much wider range here to encompass both successful and failed population replacement strategies. The black dashed line describes the total number of cases in the absence of any interventions, the solid black line represents the cumulative incidence in a model where population replacement is represented using a phenomenological description of the decline in vector competence (model (1) of the main text), and the dashed grey line represents the predicted cumulative incidence using model (S3-1) that tracks mosquitoes with and without the symbiont. As in Supplementary Information S2, the initial *Wolbachia* prevelance is set at 2%.

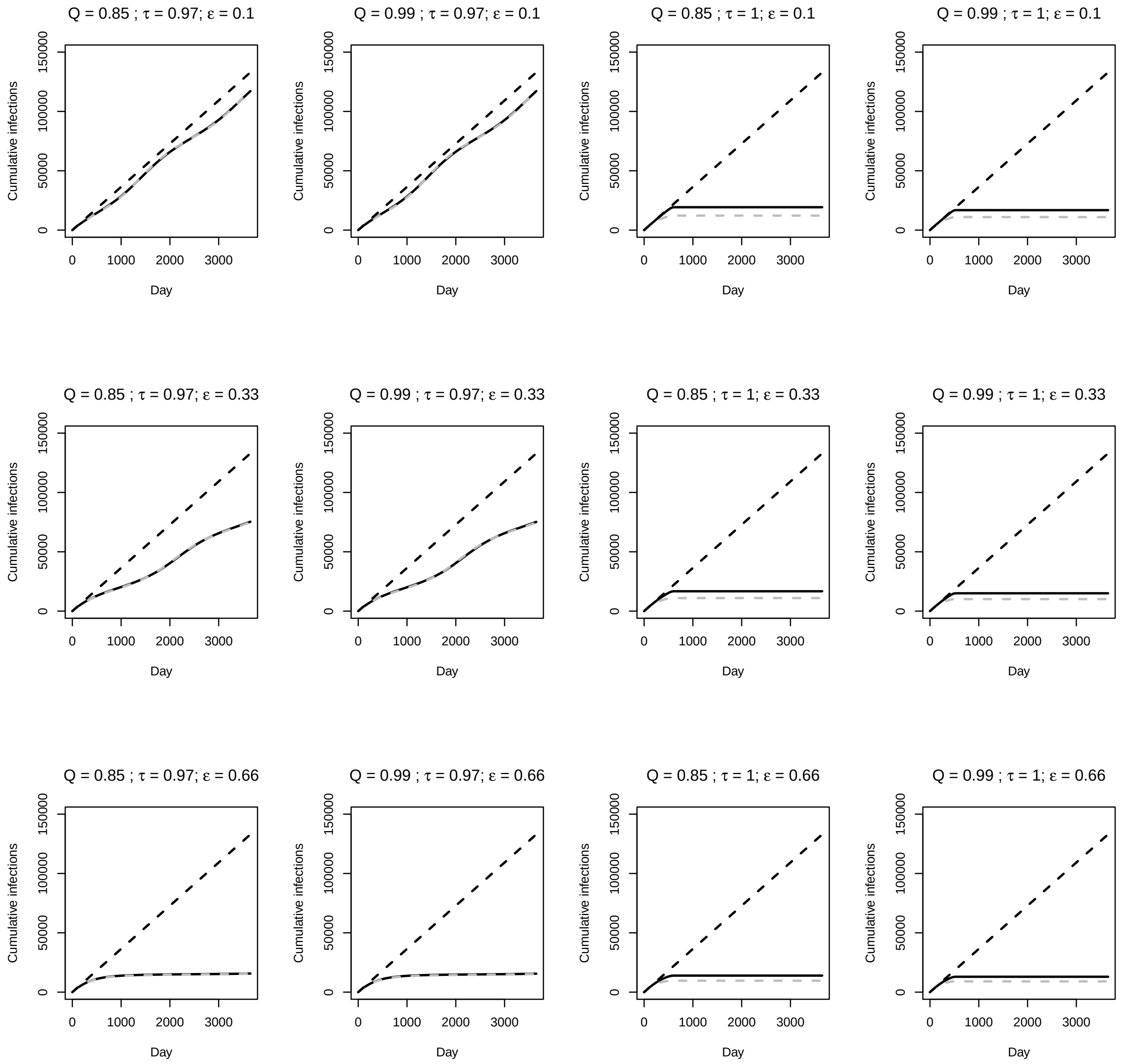

Figure B. The effects on cumulative incidence for model (S3-1) which combines attempted *Wolbachia* introgression with a partially effective vaccine program. The black, dashed line describes the total number of cases in the absence of any interventions, the solid black line represents the same quantity for model (1) in the main text, and the dashed grey line represents the predicted cumulative incidence using model (S3-1) that tracks the dynamics of mosquitoes with and without the symbiont. The vaccination fraction increases from the top row to the bottom row, and the ability of *Wolbachia* to spread is varied across columns.

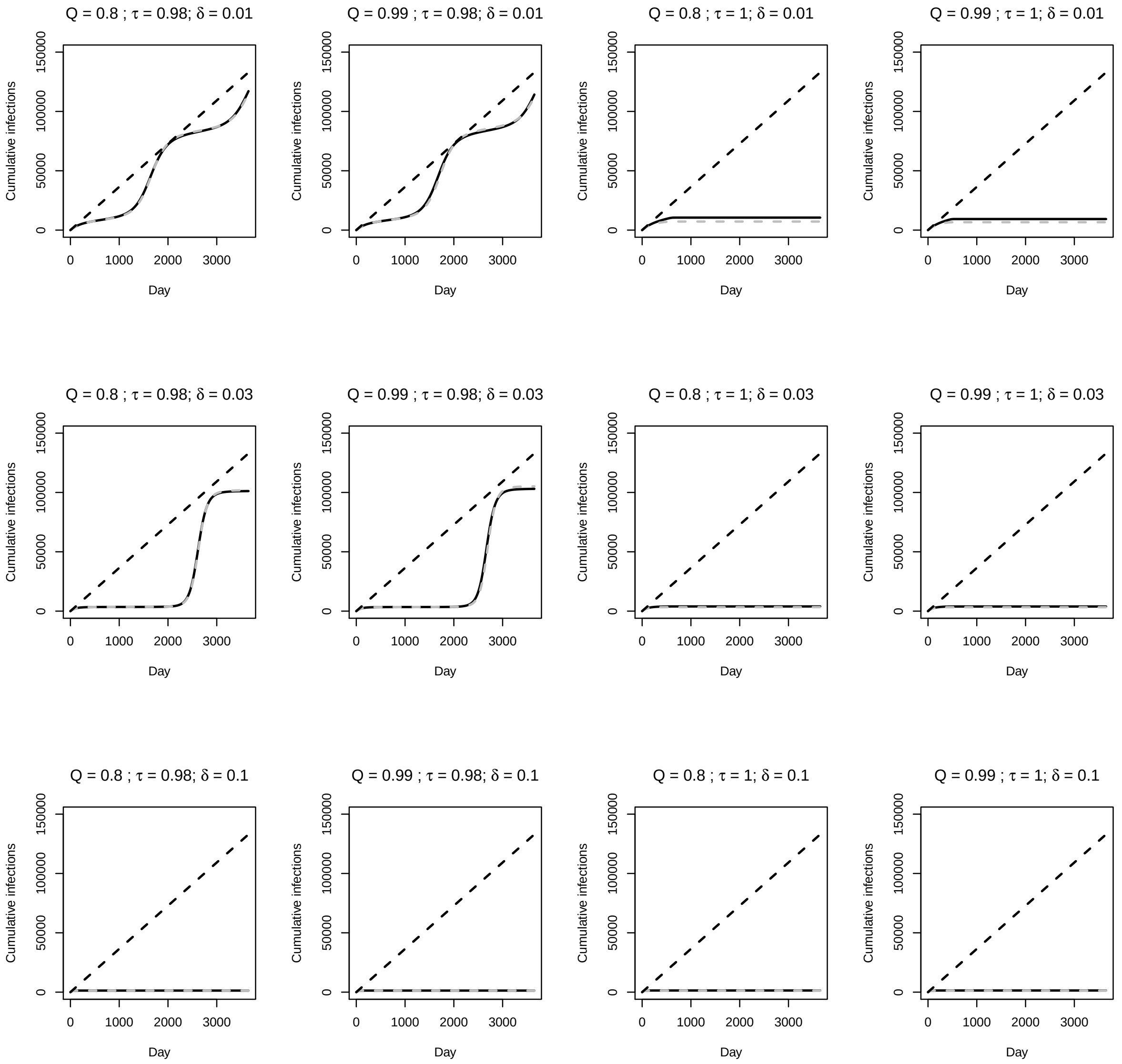

Figure C. The effects on cumulative incidence for model (S3-1) which combines attempted *Wolbachia* introgression with administration of an antimicrobial drug. The black, dashed line describes the total number of cases in the absence of any interventions, the solid black line represents the same quantity for model (1) in the main text, and the dashed grey line represents the predicted cumulative incidence using model (S3-1) that tracks mosquitoes with and without the symbiont. The drug-induced recovery rate increases from the top row to the bottom row, and the ability of *Wolbachia* to spread is varied across columns.

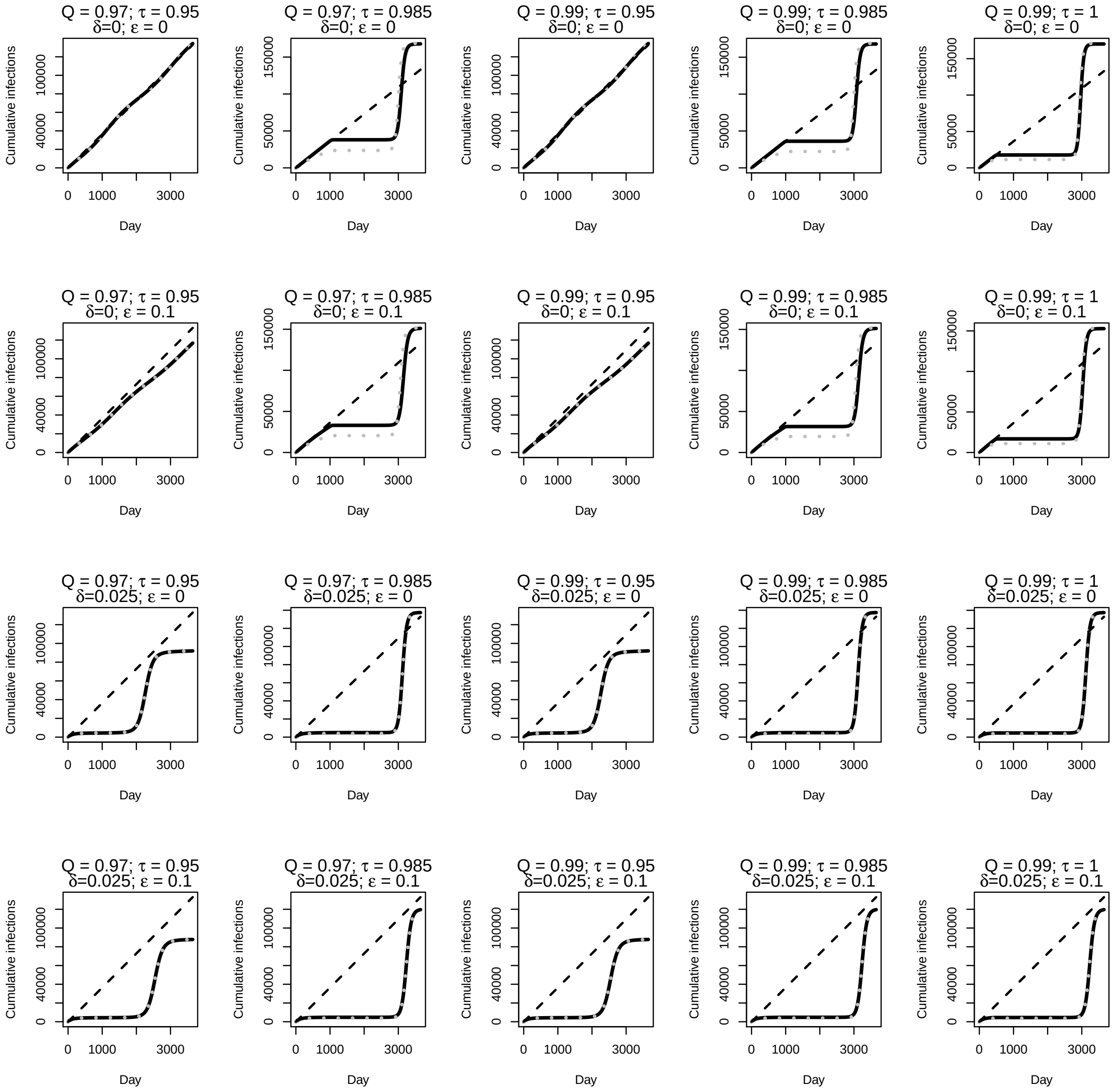

Figure D. The effects on cumulative incidence for model (S3-2) which combines attempted *Wolbachia* introgression with administration of an antimicrobial drug, and where pathogen resistance to *Wolbachia* can subsequently evolve. As in the main text, both models assume that resistance arises in a single infectious vector after $\tau_R = 2/3T_H$ days. The first row describes the epidemiological effect of *Wolbachia* spread in the absence of any clinical intervention, the second row describes the dynamics when only a vaccine is combined with population replacement, the third row describes the dynamics when only an antimicrobial is combined with population replacement, and the fourth row describes what happens when an antimicrobial is used in conjunction with a vaccine. The black, dashed line describes the total number of cases in the absence of any interventions, the solid black line represents the same quantity for model (1) in the main text, and the dashed grey line represents the predicted cumulative incidence using model (S3-2) that tracks mosquitoes with and without the symbiont.
