# Supplementary material for: Integrating Transgenic Vector Manipulation with Clinical Interventions to Manage Vector-Borne Diseases
Source: PLoS Comput Biol. 2016 Mar 10;12(3):e1004695. doi: 10.1371/journal.pcbi.1004695 (PMC4786096; doi:10.1371/journal.pcbi.1004695)
Supplement: S4 Text — (PDF) [file pcbi.1004695.s004.pdf]

Supplementary Information S4: The long-term effect of integrating transgenic population replacement with clinical interventions when relative vector competence cannot be lowered beyond a fixed threshold

Our phenomenological model $G(t) = 1/(1 + \exp(\alpha t + \beta))$ is based on the population genetic models considered in Supplementary Information S2 and S3, and also predicts long-term vector competence to eventually decline to zero as $t \to \infty$. In practice, even complete vector population replacement may not be able to reduce vector competence to zero. For instance, an anti-pathogen construct may confer only partial pathogen resistance, and thus even when the transgenic construct is at fixation, $G(t) \to \xi > 0$ as $t \to \infty$. Here we assess how a non-zero, long term reduction ξ in relative vector competence affects disease incidence at the endemic equilibrium and the effective type reproductive number T_E. As we note in the main text, the predicted patterns (Fig. A) are very similar to the case where transgenic population reduction is unable to decrease vector population recruitment to low levels (Fig. 3 of the main text).

References

Figures

Figure A. The long-term epidemiological effects of integrating clinical interventions with transgenic population replacement. The vertical axis varies the magnitude of ξ, which represents the long-term reduction in vector competence below which the vector population cannot be driven. (a) The prevalence (fraction of hosts infectious) at equilibrium after combined vaccination and transgenic population replacement relative to prevalence before the interventions begin (i.e., at the equilibrium in the absence of intervention), and (b) the corresponding effective type reproductive number at the new equilibrium resulting from the intervention in (a), both in the absence of anti-microbial medications. Panels (a-b) illustrate how absent transgenic manipulation long-term pathogen elimination is only possible in our model if the fraction of hosts vaccinated exceeds approximately 0.78, which corresponds to roughly $1 - 1/T$ (e.g., ref. [85] of the main text) for the parameter values in Table 1. A similar threshold exists for transgenic population replacement without vaccination. (c-d) present analogous results assuming vaccination and transgenic population replacement are combined with an anti-microbial medication that removes infectious hosts at the same rate as the background recovery rate, while (E-F) describe the results when an anti-microbial medication that removes infectious hosts at twice the background recovery rate is used.

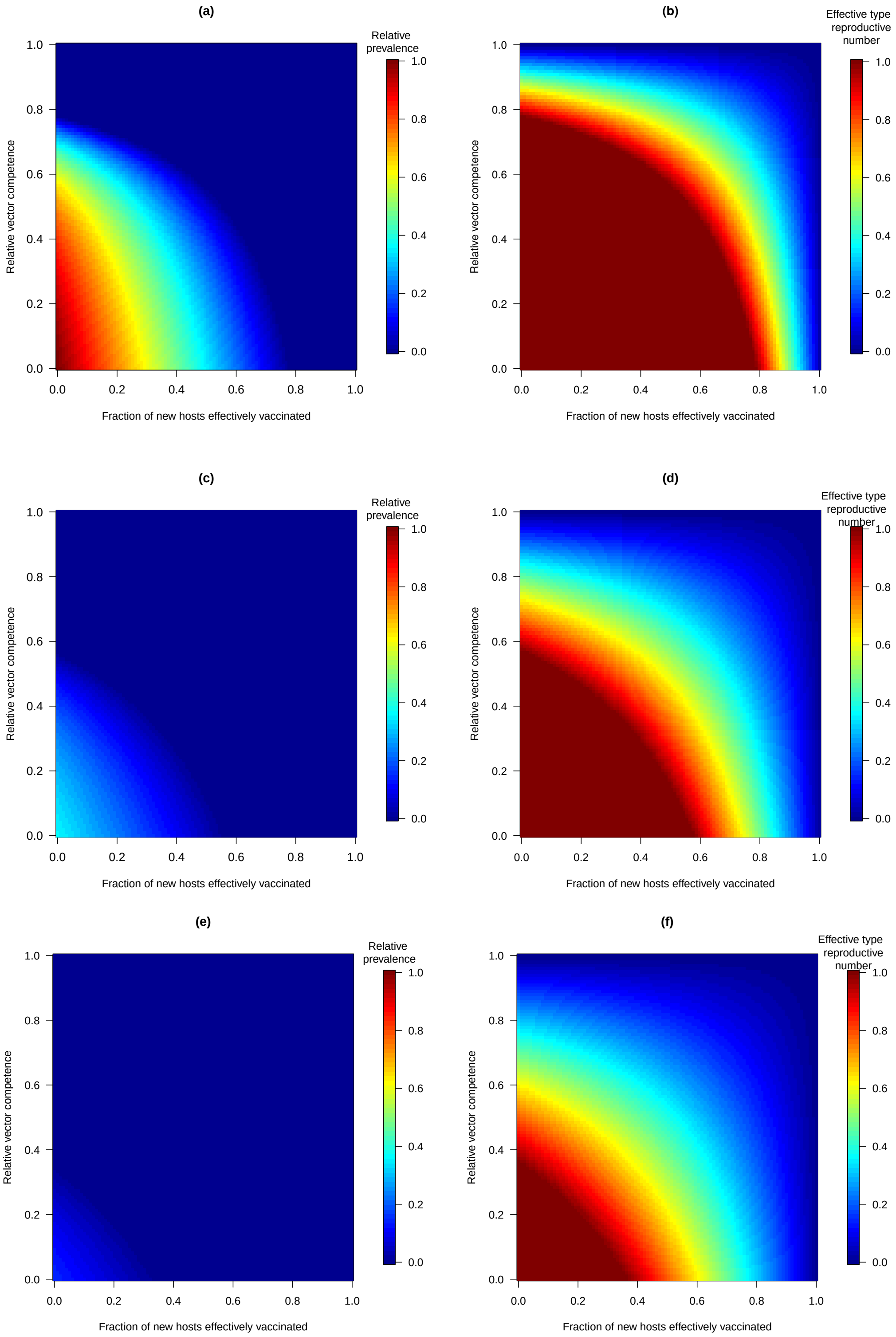
(a)
Relative prevalence
Relative vector competence
Fraction of new hosts effectively vaccinated
(b)
Effective type reproductive number
Relative vector competence
Fraction of new hosts effectively vaccinated
(c)
Relative prevalence
Relative vector competence
Fraction of new hosts effectively vaccinated
(d)
Effective type reproductive number
Relative vector competence
Fraction of new hosts effectively vaccinated
(e)
Relative prevalence
Relative vector competence
Fraction of new hosts effectively vaccinated
(f)
Effective type reproductive number
Relative vector competence
Fraction of new hosts effectively vaccinated
